# Identification of a CD44-dependent control of astrocytic autophagic activity in Alzheimer’s disease

**DOI:** 10.1016/j.tjpad.2026.100601

**Published:** 2026-05-20

**Authors:** Haiyan Wang, Ying Long, Yu Tang, Lijie Duan, Zijie Wang, Shuzhen Zhang, Yanqing Yin, Jiawei Zhou, Wenjuan Wu, Chunjiu Zhong

**Affiliations:** a Department of Neurology, Zhongshan Hospital, Fudan University, Shanghai, China; b State Key Laboratory of Brain Function and Disorders, Institutes of Brain Science, Fudan University, Shanghai, China; c National Clinical Research Center for Aging and Medicine, Huashan Hospital, Fudan University, Shanghai, China; d Institute of Neuroscience, State Key Laboratory of Neuroscience, CAS Center for Excellence in Brain Science and Intelligence Technology, Chinese Academy of Sciences, Shanghai, China; e China Resources WITest (Shanghai) Medical Instruments Co., Ltd, Shanghai, China; f Department of Laboratory Medicine, Shanghai East Hospital, Tongji University School of Medicine, Shanghai, China; g Department of Neurology, Jinshan Hospital Affiliated to Fudan University, Shanghai, China

**Keywords:** Alzheimer's disease, CD44, Astrocytes, Autophagy, Transcriptomic

## Abstract

**Background:**

Alzheimer's disease (AD) is a progressive neurodegenerative disorder characterized by cognitive decline and memory impairment. Despite extensive research, the precise molecular mechanisms driving AD pathogenesis remain incompletely understood. This study sought to identify robust molecular targets and cellular basis underlying AD progression.

**Methods:**

We performed a systematic analysis of cross-regional transcriptomic datasets from AD patients, integrating differential expression analysis across 14 Gene Expression Omnibus (GEO) datasets with cross-regional intersection mapping. Single-nucleus RNA sequencing (snRNA-seq) was employed to resolve cell-type-specific expression patterns. Furthermore, cellular communication analysis and functional enrichment of astrocyte-specific genes were conducted. The biological role of the identified candidate was validated in vitro using Aβ42 oligomer-treated primary astrocytes via siRNA-mediated knockdown and plasmid-driven overexpression, with autophagic activity assessed through LC3-II and p62 expression.

**Results:**

The transmembrane glycoprotein receptor CD44 was identified as consistently upregulated across AD-vulnerable brain regions, including the temporal cortex, frontal cortex, entorhinal cortex, and hippocampus. snRNA-seq analysis identified this upregulation primarily to astrocytes. Intercellular signaling analysis indicated that the CD44-SPP1 axis enhanced astrocyte-glial crosstalk. Functional enrichment analysis linked astrocytic CD44 to the modulation of autophagy pathways. In vitro experiments demonstrated that CD44 knockdown promoted autophagic activation (increased LC3-II and decreased p62), whereas CD44 overexpression suppressed autophagic activity.

**Conclusion:**

Our findings establish CD44 as a pivotal regulator of astrocytic autophagy in AD, highlighting its potential as a novel therapeutic target.

## 1 Introduction

Alzheimer's disease (AD), the leading cause of dementia, is a progressive neurodegenerative disorder defined pathologically by the accumulation of extracellular amyloid-β (Aβ) plaques and intracellular neurofibrillary tangles of hyperphosphorylated tau (p-tau) [1,2]. The global burden of AD is escalating at an alarming rate; projections suggest that by 2060, the number of affected individuals will reach 13.8 million in the United States and approximately 49.89 million in China [3,4]. Current pharmacological interventions, such as cholinesterase inhibitors and NMDA receptor antagonists, offer only transient symptomatic relief without altering the underlying disease trajectory [5]. While recently FDA-approved disease-modifying therapies (DMTs), including the monoclonal antibodies lecanemab [6] and donanemab [7], show promise in slowing cognitive decline, their efficacy is modest and often accompanied by risks of amyloid-related imaging abnormalities (ARIA). These limitations underscore an urgent requirement for novel therapeutic targets that address the multifaceted pathological mechanisms beyond the amyloid cascade. The prolonged preclinical phase of AD, where molecular pathology precedes clinical symptoms by decades, presents a critical window for early intervention [8]. Identifying diagnostic biomarkers is therefore essential, yet a distinction must be made between markers of association and drivers of pathogenesis [9,10].

Integrated omics analysis of multi-cohort data has emerged as a robust strategy for identifying high-confidence targets. Previous transcriptomic studies have stratified AD into molecular subtypes based on synaptic [11], metabolic [12], or mitochondrial dysfunction [13]. Sex-based dimorphisms have also been observed, with female AD patients exhibiting more pronounced deficits in synaptic regulation, neurotransmitter pathways (e.g., glutamate and GABA), Aβ aggregation, and protein folding [14]. However, traditional bulk transcriptomics lacks the resolution to distinguish cell-type-specific contributions to disease progression.

CD44, a multifunctional transmembrane glycoprotein, facilitates cell-cell and cell-matrix interactions and has been implicated in the pathogenesis of various neurodegenerative diseases, including Parkinson’s disease (PD) and amyotrophic lateral sclerosis [15,16]. Nevertheless, the specific role of CD44 in AD remains poorly defined. Concurrently, autophagy, a conserved lysosomal degradation pathway essential for maintaining cellular homeostasis, is known to be impaired in AD, contributing to the accumulation of toxic protein aggregates [17,18]. While neuronal autophagy is well-documented, the impact of astrocytic autophagic dysfunction on central nervous system (CNS) homeostasis is increasingly recognized as a vital component of neurodegeneration [19].

In the present study, we utilized a cross-regional transcriptomic analysis to identify CD44 as a high-confidence hub gene in AD. We demonstrate that CD44 is specifically upregulated in astrocytes during AD. Using a combination of bioinformatics and experimental validation, we show that CD44 modulates astrocytic autophagic activity, evidenced by changes in the gold-standard markers LC3-II and p62 [20]. Our results suggest that astrocytic CD44 contributes to AD pathology by suppressing autophagic activity, thereby nominating it as a viable target for therapeutic intervention.

## 2 Materials and methods

### 2.1 Data acquisition

Fourteen transcriptomic datasets (microarray and RNA-seq) and two snRNA-seq datasets from post-mortem brain tissues of AD patients and non-demented controls were obtained from the Gene Expression Omnibus (GEO) database. Human brain transcriptomic datasets were identified through systematic searches of GEO database using the keywords 'Alzheimer's disease', 'brain', and 'Homo sapiens'. Inclusion criteria were: (1) post-mortem brain tissues from AD patients and non-demented controls; (2) availability of raw or processed gene expression data; (3) sample size ≥ 5 per group. This search yielded fourteen independent datasets spanning multiple brain regions (frontal cortex, temporal cortex, entorhinal cortex, and hippocampus) and profiling platforms (microarray and RNA-seq). Key characteristics were summarized in Table 1. Platform annotation files were used to map microarray probes to official gene symbols. A complete list with GEO accession numbers, demographic and clinicopathological characteristics was provided in Supplementary Table S1.

**Table 1:** Information of RNA-seq and snRNA-seq datasets enrolled in the study.

	GEO ID	Donor	Tissue region	Experiment
1	GSE29378	31 AD, 32 control	Hippocampus	Microarray
2	GSE1297	22 AD, 9 control	Hippocampus	Microarray
3	GSE33000	310 AD, 157 control, 157 HD	Prefrontal cortex	Microarray
4	GSE132903	97 AD, 98 control	Middle temporal gyrus	Microarray
5	GSE122063	56 AD, 44 control, 36 VaD	Frontal cortex, temporal cortex	Microarray
6	GSE118553	52 AD, 27 control, 33 asymAD	Temporal cortex, cerebellum, frontal cortex, entorhinal cortex	Microarray
7	GSE48350	80 AD, 173 Control	Entorhinal cortex, hippocampus, postcentral gyrus, superior frontal gyrus	Microarray
8	GSE44772	129 AD, 101 control	Cerebellum, dorsolateral prefrontal cortex, occipital cortex	Microarray
9	GSE36980	32 AD, 47 control	Frontal cortex, temporal cortex, hippocampus	Microarray
10	GSE84422	328 AD, 214 control	Temporal cortex, frontal cortex, occipital cortex, hippocampus	Microarray
11	GSE5281	87 AD, 74 control	Temporal cortex, frontal cortex, occipital cortex, entorhinal cortex, hippocampus	Microarray
12	GSE53697	9 AD, 8 control	Frontal cortex	RNA-seq
13	GSE125583	219 AD, 70 control	Fusiform gyrus	RNA-seq
14	GSE95587	84 AD, 33 control	Fusiform gyrus	RNA-seq
15	GSE188545	6 AD, 6 control	Middle temporal gyrus	snRNA-seq
16	GSE174367	11 AD, 8 control	Prefrontal cortex	snRNA-seq

### 2.2 Differential expression and intersection analysis

Differential expression analysis was executed using the limma package (v3.52.4) in R (v4.1.0) [21]. To ensure robustness, transcripts with low abundance (detected in ≤ 50% of samples) were excluded. Differentially expressed genes (DEGs) were defined by the thresholds of *p*-value < 0.05 and a fold-change (|FC| ≥ 1.5). Heatmap and hierarchical clustering were visualized using the pheatmap package (v1.0.12). Cross-regional intersections were visualized via the UpSetR package (v1.4.0) [22] to identify conserved molecular signatures across distinct anatomical domains.

### 2.3 Functional and pathway enrichment analysis

Gene Ontology (GO) and Kyoto Encyclopedia of Genes and Genomes (KEGG) pathway enrichment analysis were conducted using clusterProfiler package (v4.6.2) [23]. Reactome pathway analysis was performed with the ReactomePA package (v1.48.0) [24]. Terms and pathways with an adjusted *p*-value < 0.05 were considered significant and visualized using bar or bubble plots.

### 2.4 Protein-protein interaction (PPI) network and hub gene prioritization

PPI networks were retrieved from the STRING database (confidence score > 0.4) and modeled using Cytoscape (v3.10.2) [25]. To identify topologically significant nodes, we applied the Maximum Clique Centrality (MCC) algorithm within the cytoHubba plugin [26]. MCC calculates centrality by enumerating all maximal cliques in the network and assigning scores based on a gene's participation in these cliques. Genes appearing in more and larger cliques receive higher MCC scores, reflecting their potential importance as network hubs. The MCC method was selected for its superior performance in identifying essential proteins within complex biological networks compared to standard centrality measures.

### 2.5 Convergent functional genomic (CFG) ranking

Hub genes were prioritized using the CFG framework via the AlzData database [27]. Genes were scored based on: (1) association with both GWAS (*p* < 0.001) and eQTL (*p* < 0.001); (2) GWAS association alone (*p* < 0.001); (3) physical interaction with core AD genes (APP, PSEN1, PSEN2, APOE, or MAPT; *p* < 0.05); (4) differential expression in pre-pathology AD mouse models; (5) correlation with Aβ or tau pathology in mouse models (cor: correlation coefficient; ns: *p* > 0.05, *p < 0.05, **p < 0.01, ***p < 0.001). Total scores ranged from 0 to 5.

### 2.6 snRNA-seq data processing and cell type annotation

snRNA-seq data were processed using the Seurat package (v5.1.0) [28]. Cells with less than 300 genes or higher than 8000 genes, or mitochondrial gene ratio higher than 2%, or predicted doublets were excluded. Data were normalized, scaled, and clustered using the top 20 principal components. Cell types were identified with the FindClusters function (resolution = 0.1) and visualized in two dimensions using uniform manifold approximation and projection (UMAP). Major cell types were annotated based on canonical marker genes curated from the literature and the CellMarker 2.0 database. The following marker genes were used for each major cell type: Astrocytes (GFAP, AQP4, ALDH1L1, SLC1A3, SLC1A2), Excitatory neurons (SLC17A7, NRGN, BCL11B, NEUROD1, NEUROD2), Inhibitory neurons (GAD1, GAD2, CCK, CALB2, SST), Microglia (P2RY12, TMEM119, CX3CR1, CSF1R, AIF1), Oligodendrocytes (MBP, PLP1, MOG, OLIG2, MAG), OPCs (PDGFRA, SOX10, VCAN, CSPG4, MEGF11), Endothelial cells (CLDN5, PECAM1, VWF, FLT1, CD34), Pericytes (PDGFRB, RGS5, ABCC9, KCNJ8, COL3A1).

### 2.7 Intercellular communication analysis

Cell-cell communication was inferred from snRNA-seq datasets (GSE188545 and GSE174367) using the CellChat package (v2.2.0.90) [29]. The statistical framework implemented in CellChat is based on a permutation test that assesses the significance of each ligand-receptor interaction. Specifically, CellChat first computes a communication probability for each ligand-receptor pair between each pair of cell groups using a law of mass action-based model that integrates: expression levels of ligands in sender cells, expression levels of receptors in receiver cells, effects of co-receptors, agonists, and antagonists. Statistical significance is then assessed via permutation testing. The null hypothesis is that the observed communication probability could occur by chance given the gene expression distribution across cells. We used the default setting of nboot = 100 permutations for each ligand-receptor pair. Cell labels were randomly permuted across cells while preserving the original cell group sizes. For each permutation, communication probabilities were recalculated for all ligand-receptor pairs. The FDR-adjusted *p*-value for each interaction was derived as the proportion of permutations in which the permuted communication probability equaled or exceeded the observed probability. An interaction was considered statistically significant if the adjusted *p*-value < 0.05 after multiple testing correction. A ligand or receptor was considered "highly expressed" if its truncated mean expression value was in the top 25th percentile of all expressed genes in that cell type. Besides, the LIANA, a comprehensive combination of ligand-receptor methods and resources, were further confirmed using the liana package (v0.1.14) [30]. The preferentially highly-ranked interactions were generated from the interaction rankings of the algorithms underlying natmi, connectome, logfc, sca, and cellphonedb. The smaller the value of aggregate rank, the higher the ranking of the ligand-receptor pair.

### 2.8 Primary astrocyte culture

All procedures were approved by the Institutional Ethics Committee of Zhongshan Hospital, Fudan University. Primary astrocytes were isolated from P0 C57BL/6 mouse cortices [31]. Briefly, dissected brain tissues were washed, mechanically dissociated, and centrifuged at 300 g for 3 min. The cell pellet was resuspended in DMEM medium supplemented with 10% fetal bovine serum (FBS) and 1% penicillin/streptomycin (P/S) and seeded into poly-l-lysine-coated T75 flasks. After 2 days in culture, microglia and other floating cells were removed by moderate shaking, achieving ≥ 95% astrocyte purity.

### 2.9 CD44 knockdown and overexpression

CD44 was knocked down using a specific siRNA (siCD44) and a non-targeting control (siNC) (SynBio Co., Ltd). The siRNA sequences were as follows: siCD44 sense 5′-CGAUGCUUCAAACAUUAUATT-3′, antisense 5′-UAUAAUGUUUGAAG CAUCGTT-3′; siNC: sense 5′-UUCUCCGAACGUGUCACGUTT-3′, antisense 5′-ACGUGACACGUUCGGAGAATT-3′ For CD44 overexpression, the pCMV3-C-HA plasmid containing the full-length CD44 cDNA (CD44) (#HG12211-CY, Sino Biological) was used, with an empty vector serving as the control (GFP). Transfection efficiency was validated by western blotting.

### 2.10 Aβ42 oligomer treatment

Aβ42 oligomers (Aβ) were prepared as previously described [32]. Briefly, synthetic Aβ42 peptide was monomerized using hexafluoroisopropanol (HFIP), dissolved in anhydrous DMSO, and then diluted in cold DF12 culture medium to a stock concentration of 500 µM. The solution was incubated at 4 °C for 48 h to form oligomers. After siRNA or plasmid transfection for 48 h, the astrocyte culture medium was replaced with DMEM containing 5% FBS and 1% P/S, followed by treatment with 5 μM Aβ to mimic AD-related toxicity.

### 2.11 RNA sequencing and analysis

Total RNA was extracted using the RNeasy Mini Kit (QIAGEN) according to the manufacturer’s instructions. RNA quality and integrity were assessed using a Qubit 3.0 Fluorometer and an Agilent 4200 Bioanalyzer. Libraries were prepared with the VAHTS Universal V10 RNA-seq Library Prep Kit (Vazyme, #NR606) and sequenced on an Illumina NovaSeq 6000. Raw FASTQ files were processed as described previously [33]. Adapters and low-quality bases were trimmed, and the clean reads were aligned to the GRCm39 reference genome using HISAT [34]. Gene expression quantification was performed using SAMtools [35] and StringTie [36].

### 2.12 Western blotting

Cells were lysed in RIPA buffer (Thermo Fisher Scientific, #89901) supplemented with protease and phosphatase inhibitor cocktail (Thermo Fisher Scientific, #78442). Proteins were separated by SDS-PAGE, transferred to PVDF membranes, and probed with primary antibodies against β-actin (Sigma-Aldrich, #A5441), GAPDH (SAB, #1336), CD44 (Proteintech, #30854-1-AP), LC3 (MBL, #PM036), p62 (MBL, #PM045), IL1β (Santa Cruz, sc7884) or TNFα (Abcam, ab9739). After washing, membranes were incubated with HRP-conjugated secondary antibodies (Jackson ImmunoResearch: goat anti-rabbit IgG #111-035-003, goat anti-mouse IgG #115-035-003). Blots were visualized using the ECL Substrate (Thermo Fisher Scientific, #32106) on an ImageQuant LAS-4000 imaging system (Fujifilm).

### 2.13 Quantitative RT-PCR (qRT-PCR)

qRT-PCR was conducted as previously described [31]. The primers were designed and synthesized by Sangon Biotech Co., Ltd (Shanghai, China). β-Actin was used as an internal control gene. The primer sequences were as follows: Atg12, forward, 5′- CGGACCATCCAAGGACTCATTGAC-3′, reverse, 5′-TGGGGAAGGGGCAAAGGACT G-3′; Castor2, forward 5′-GCCACATCCGCTTCTTCTCCTTC-3′, reverse, 5′-TAGCAAGTGACTCGGGAACCTCTG-3′; Prkaa1, forward, 5′-AACCTGAGAACGTCCTGCTTGATG-3′, reverse, 5′-TGACTTCTGGTGCGGCATAATTGG-3′; Slc7a5, forward, 5′-GTGTGCGGCGTC TTCTCCATC-3′, reverse, 5′-ACCTCCAGCATGTAGGCGTAGTC-3′; Tuba1b, forward, 5′-CGGCTCTCTGTGGATTACGGAAAG-3′, reverse, 5′-TGGTGTGGGTGGTGAGGATGG-3′; Tubb4b, forward, 5′-GATGGCTGCCTGTGATCCAAGAC-3′, reverse, 5′-GCATCTGTTCGT CCACCTCCTTC-3′; Ulk1, forward, 5′-TTCAGCACCAGCCGCATTACG-3′, reverse, 5′-CA AAGCCAGCAGAGGGAGCAATC-3′.

### 2.14 Statistical analysis

All statistical analysis and graphical generation were performed using R (v4.1.0) and GraphPad Prism (v9.5.0). Cellular experiments included ≥ 3 biological replicates. The normal distribution of the data was confirmed by the Shapiro-Wilk test. For comparisons between two groups, we used a two-tailed *t*-test with false discovery rate (FDR) correction. The adjusted *p*-value < 0.05 was considered statistically significant. The effect size Cohen's d and 95% confidence interval (95% CI) were also calculated. For comparisons between neuropathological characteristics, we performed analysis of covariance (ANCOVA) by the aov package, using age and sex as covariates, with the effect size partial η² (eta-squared) and 95% CI reported. For correlation analysis between CD44 expression and neuropathological characteristics, Spearman's rank correlation coefficient was used. The effect size ρ (rho) and 95% CI were also reported.

## 3 Results

### 3.1 Identification of cross-regional DEGs in AD

**Fig. 1:**
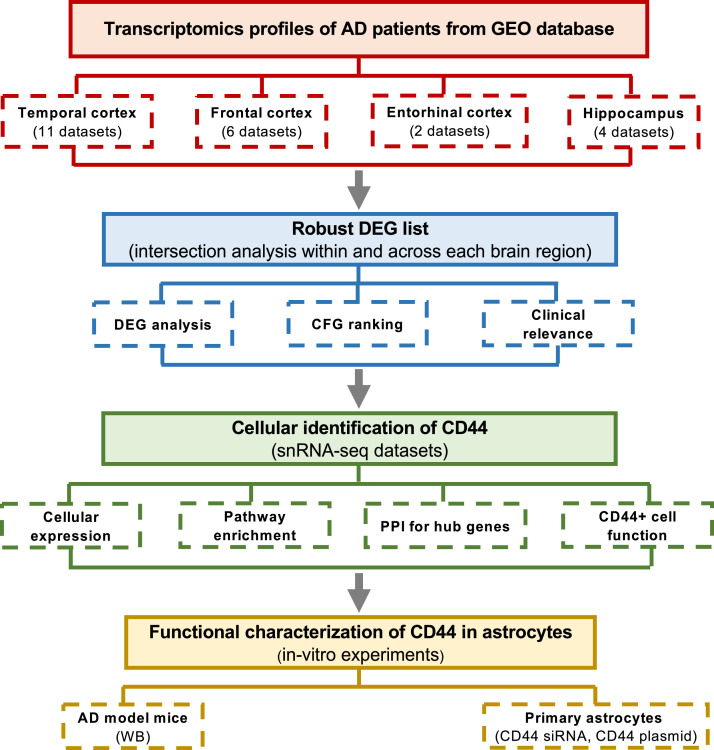
**Study workflow.** We analyzed transcriptomic profiles from four major AD brain regions, identified DEGs, and performed cross-regional intersection. High-confidence genes were prioritized using multi-evidence integration and PPI network analysis. Cellular localization and function of CD44 were investigated using snRNA-seq and in vitro experiments.

We first determined whether the transcriptomic datasets collected from different tissue regions could be integrated for analysis. To this end, we analyzed a total of 14 transcriptomic (microarray and RNA-seq) and 2 snRNA-seq datasets from multiple brain regions of AD patients and non-demented controls. Detailed information on all 16 datasets used, including GEO accession numbers, cohort demographics, brain regions, and experimental methods, was provided in Table 1. The demographic and pathological characteristics of the subjects were provided in Supplementary Table S1. The overall study design was illustrated in Fig. 1.

**Fig. 2:**
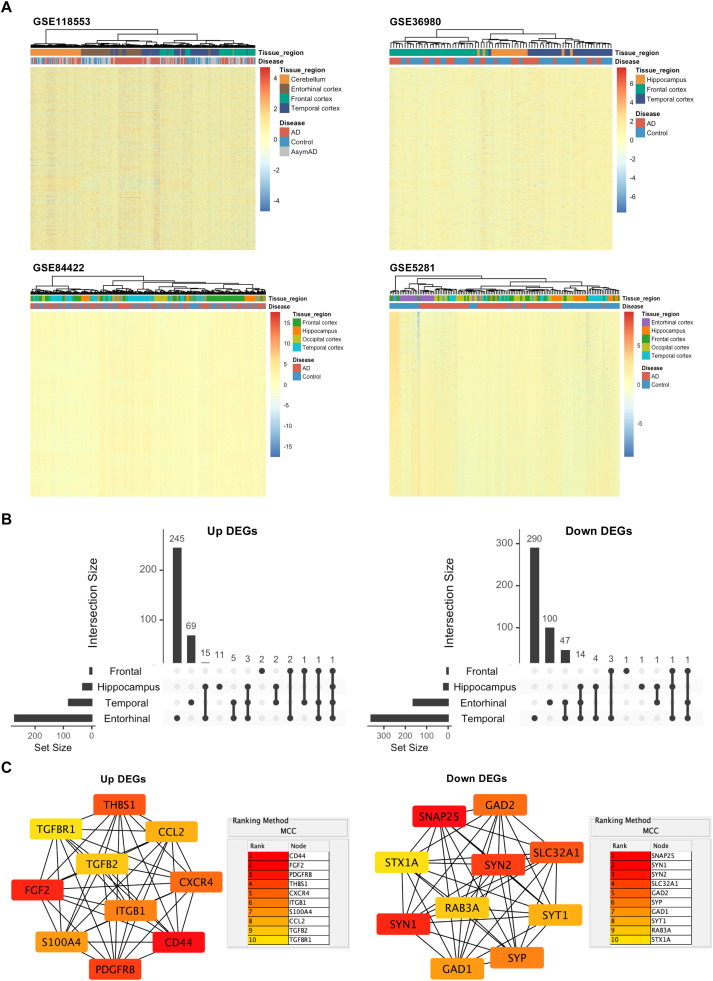
**Intersection of DEGs across four major brain regions. A** Hierarchical clustering of gene expression profiles from four datasets (GSE118553, GSE36980, GSE84422, GSE5281), showing clustering by brain region. **B** Upset plots of upregulated and downregulated DEGs across temporal cortex, frontal cortex, entorhinal cortex, and hippocampus. **C** Hub genes analyzed in Cytoscape with MCC algorithm.

Fig. 2A provided representative heatmap plots showing the four selected brain regions across multiple datasets. These datasets were chosen to illustrate regional heterogeneity (prefrontal cortex, hippocampus, entorhinal cortex, temporal cortex). Hierarchical clustering revealed that transcriptomic profiles clustered primarily by brain region rather than disease status, highlighting the importance of conducting differential expression analysis in a region-specific manner, as pooling datasets from distinct anatomical regions may obscure genuine gene expression patterns. An intersection analysis of DEGs was performed across multiple datasets within the same brain region. DEGs consistently identified in more than half of the datasets for a given region were retained for subsequent analysis. Matrix plots were generated to visualize the distribution of intersected upregulated and downregulated DEGs across four major brain regions: temporal cortex, frontal cortex, entorhinal cortex, and hippocampus (Supplementary Fig. S1). We identified intersected DEGs (in more than two regions) for the temporal cortex (82 up, 360 down), frontal cortex (7 up, 6 down), entorhinal cortex (272 up, 163 down), and hippocampus (32 up, 21 down) (Table 2).

**Table 2:** Distribution of DEGs in four brain regions.

Tissue region	Number of datasets	Total DEGs		Intersected DEGs (≥ half of datasets)	
		Up	Down	Up	Down
Temporal cortex	8	10,054	14,957	82	360
Frontal cortex	8	3024	9297	7	6
Entorhinal cortex	4	4118	4162	272	163
Hippocampus	6	5190	9221	32	21

To identify high-confidence candidates, we next conducted a cross-tissue intersection analysis of DEGs from the four brain regions (Fig. 2B). Among the upregulated DEGs, CD44 emerged as a ubiquitous upregulated DEG across all vulnerable neuroanatomical regions, validated by non-parametric ANCOVA analysis (Supplementary Table S2); GFAP across temporal cortex, frontal cortex, and entorhinal cortex; SYTL4, WWTR1, and APLNR across temporal cortex, entorhinal cortex, and hippocampus, indicating their potential roles in a core AD pathway prevalent across neuroanatomical regions. Among the downregulated DEGs, RPH3A was shared across temporal cortex, frontal cortex, and entorhinal cortex; SST across temporal cortex, frontal cortex, and hippocampus; 14 genes (CHGB, SYN2, GABRG2, NEFL, RGS4, VSNL1, CPLX1, INA, ENC1, HPRT1, NEFM, SCN3B, STMN2, and SV2B) across temporal cortex, entorhinal cortex, and hippocampus, implicating a systematic impairment of neuronal integrity in AD pathogenesis.

To determine the key genes in these pan-tissue DEGs, PPI network analysis was used. It was revealed that the top 10 hub genes from the upregulated DEGs were CD44, FGF2, PDGFRB, THBS1, CXCR4, ITGB1, S100A4, CCL2, TGFB2, and TGFBR1, while those from the downregulated DEGs were SNAP25, SYN1, SYN2, SLC32A1, GAD2, SYP, GAD1, SYT1, RAB3A, and STX1A (Fig. 2C). Among them, CD44 ranked as the most significant hub gene in the upregulated set, suggesting its potential central role in the network underlying AD dysfunction.

CFG analysis further supported the relevance of these hub genes with AD-related evidence (Table 3). Five genes (CD44, CXCR4, SYN1, GAD1, and SYT1) showed remarkable association with AD, achieving CFG scores ≥ 3. Of note, CD44 physically interacted with APP and PSEN1 and correlated strongly with both Aβ burden (r = 0.719) and tau pathology (r = 0.793) in AD mouse models. Consistent with this, CD44 protein levels were increased as revealed in the NeuroPro database (Table 4), indicating its potential role in AD progression.

**Table 3:** CFG ranking result for hub DEGs.

Gene	eQTL	GWAS	PPI	Early DEG	Pathology cor (Aβ)	Pathology cor (tau)	CFG
CD44	2	0	APP,PSEN1	NA	0.719,***	0.793,***	3
FGF2	5	0	PSEN2,MAPT,APOE	NA	NA	NA	2
PDGFRB	0	0	APP,PSEN2,MAPT,APOE	NA	−0.141,ns	0.003,ns	1
THBS1	2	0	PSEN2,APOE	NA	NA	NA	2
CXCR4	3	1	APP,APOE	no	0.104,ns	0.490,ns	3
S100A4	2	0	-	NA	0.347,*	0.603,*	2
CCL2	0	0	-	NA	NA	NA	0
TGFB2	2	0	APP,MAPT,APOE	no	0.080,ns	0.347,ns	2
TGFBR1	0	0	APP,PSEN1,MAPT,APOE	NA	0.860,***	0.616,*	2
SNAP25	2	0	MAPT	NA	NA	NA	2
SYN1	2	NA	-	yes	−0.413,**	−0.609,*	3
SYN2	3	0	-	yes	−0.260,ns	−0.191,ns	2
SLC32A1	0	0	-	NA	NA	NA	0
GAD2	1	0	-	NA	NA	NA	1
SYP	0	NA	-	no	−0.728,***	−0.726,**	1
GAD1	3	0	-	yes	−0.207,ns	−0.562,*	3
SYT1	0	7	MAPT	yes	−0.209,ns	−0.471,ns	3
RAB3A	0	0	-	no	−0.455,**	−0.786,***	1
STX1A	0	0	-	yes	−0.285,ns	−0.208,ns	1

PPI: Target gene has significant physical interaction with APP, PSEN1,PSEN2,APOE or MAPT (*P*-val < 0.05).

ns: *P*-val > 0.05; * *P*-val < 0.05; ** *P*-val <0.01;*** *P*-val < 0.001.

**Table 4:** CD44 protein expression in NeuroPro database.

Gene	Protein	Reference	Condition	Direction	NFT	Plaque	CAA	Location
CD44	H0YD13	Dai 2018	AD/C	Up	0	0	0	Frontal Cortex
CD44	P16070	Xu 2019	AD/C	Up	0	0	0	Cingulate Gyrus
CD44	P16070	Xu 2019	AD/C	Up	0	0	0	Cerebellum
CD44	P16070	Johnson 2020	AD/C	Up	0	0	0	Frontal Cortex
CD44	P16070	Johnson 2020	PCL/C	Up	0	0	0	Frontal Cortex
CD44	P16070	Higginbotham 2020	AD/C	Up	0	0	0	Frontal Cortex
CD44	P16070	Stepler 2020	AD/C	Up	0	0	0	Hippocampus
CD44	P16070	Wang 2020	AD/C	Up	0	0	0	Frontal Cortex
CD44	P16070	Xiong 2019	AD/C	Up	0	1	0	Hippocampus
CD44	P16070	Ping 2020	AD/C	Up	0	0	0	Frontal Cortex
CD44	P16070	McKetney 2019	AD/C	Up	0	0	0	Entorhinal Cortex
CD44	P16070	Wingo 2020	AD/C	Up	0	0	0	Frontal Cortex
CD44	P16070	Zellner 2022	AD/C	Up	0	0	0	Parietal Cortex
CD44	P16070	Johnson 2022	AD/C	Up	0	0	0	Frontal Cortex
CD44	P16070	Johnson 2022	PCL/C	Up	0	0	0	Frontal Cortex
CD44	P16070	Astillero-Lopez 2022	AD/C	Up	0	0	0	Entorhinal Cortex
CD44	P16070	Hondius 2016	AD/C	Up	0	0	0	Hippocampus
CD44	P16070	Johnson 2018	AD/C	Up	0	0	0	Frontal Cortex
CD44	P16070	Mendonca 2019	AD/C	Up	0	0	0	Parahippocampal Cortex
CD44	P16070	Mendonca 2019	AD/C	Up	0	0	0	Frontal Cortex
CD44	P16070	Xu 2019	AD/C	Up	0	0	0	Hippocampus
CD44	P16070	Xu 2019	AD/C	Up	0	0	0	Entorhinal Cortex

NFT: Neurofibrillary tangles; CAA: Cerebral amyloid angiopathy; PCL: Preclinical Alzheimer's disease.

### 3.2 Association of CD44 expression with AD clinical characteristics

**Fig. 3:**
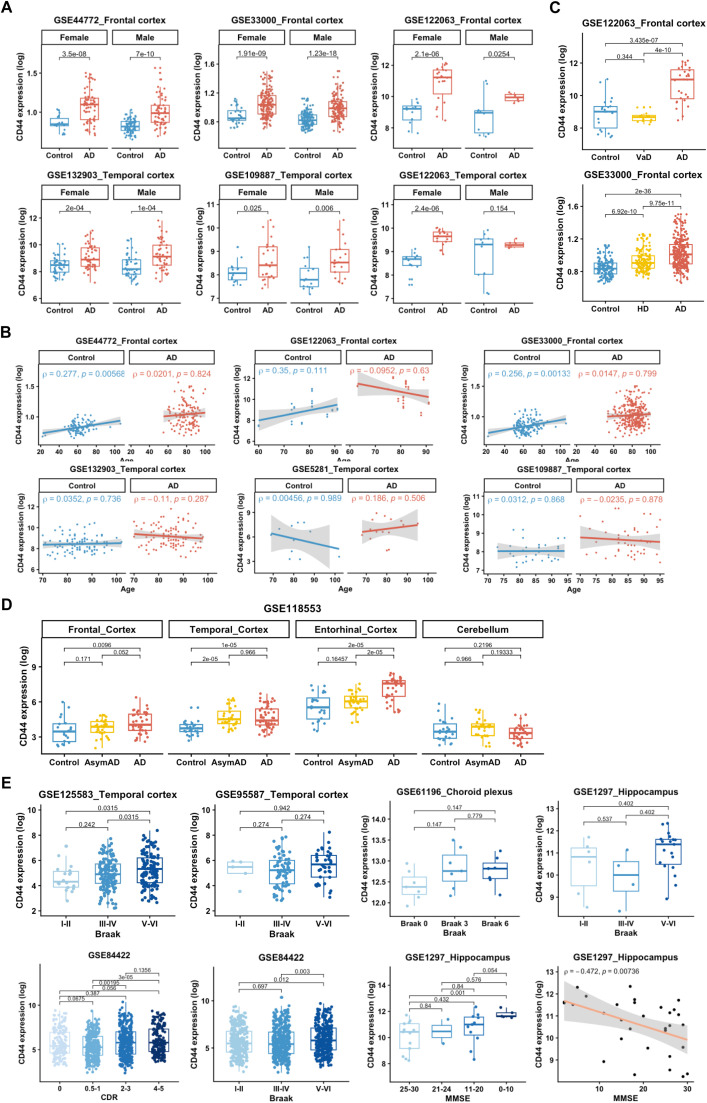
**CD44 expression and AD clinical characteristics. A** Comparison of CD44 expression levels between males and females within the AD group and the control group, respectively. **B** Pearson correlation analysis between CD44 expression levels and age in the AD and control groups. The correlation coefficient (R) is indicated. **C** CD44 expression in different severity of dementia, including MMSE, CDR and Braak. **D** CD44 expression in asymptomatic AD and AD groups. **E** CD44 expression in other dementia, including Huntington's disease (HD) and vascular dementia (VaD). Wilcoxon test and Kruskal-Wallis test were used for comparison between two groups and multiple groups, respectively. Significance levels are denoted as follows: ns, not significant; *p < 0.05; **p < 0.01; ***p < 0.001.

To evaluate the clinical relevance of CD44, we examined its expression in relation to important clinical characteristics, including sex, age, Braak stage, Mini-Mental State Examination (MMSE) score, and Clinical Dementia Rating (CDR). Overall, CD44 expression exhibited an elevated trend in females than in males in both AD and control groups (Fig. 3A), and an increased trend with age in controls, but not in AD (Fig. 3B). This phenomenon is consistent with the results from Metafun-AD resource, a web tool designed for identifying sex-specific genes relevant to AD using meta-analysis, showing that CD44 upregulation in AD was significant in cortex of females (log_2_FC = 0.255, adjusted *P* = 0.015, 95% CI [0.1075, 0.4018]), but not in that of males (log_2_FC = 0.244, adjusted *P* = 0.081, 95% CI [0.0703, 0.4183]) (Table 5). We also examined CD44 expression in other dementia and found that CD44 was elevated in Huntington's disease (HD), though to a lesser extent than in AD, but not in vascular dementia (VaD) (Fig. 3C), demonstrating a relatively high degree of specificity for AD. CD44 was significantly elevated in the neocortex (frontal, temporal, and entorhinal cortex) between AD and asymptomatic AD groups, and increased in the frontal and temporal cortex during AsymAD as compared to healthy control (Fig. 3D). Intriguingly, while CD44 expression was markedly elevated in AD, it did not exhibit a linear correlation with dementia severity (MMSE, CDR) (Fig. 3E). To explore whether CD44 upregulation is an early event associated with AD pathology, we performed a logistic regression analysis using another dataset GSE84422, which contained all the neuropathological indicators we wanted, including age, sex, tissue origin, CDR and Braak. Unfortunately, in the group of CDR = 0 (normal cognition) or CDR > 0 (impaired cognition), no obvious association was observed between higher CD44 expression and a higher Braak stage (adjusted *p*-value > 0.05) (Supplementary Table S3). Therefore, it can be concluded that the increase in CD44 is not an independent indicator for tracking pathological changes and does not represent the early pathological events of AD, but may merely be an accompanying event of neurodegeneration or dementia.

**Table 5:** Sex-based CD44 mRNA expression using meta-analysis in Metafun-AD web resource.

Gene	Brain region	Sex	log_2_FC (AD vs Control)	CI lower	CI upper	*P*.value	Adjusted *P*.value
CD44	Cortex	Female	0.255	0.108	0.402	0.001	0.015
CD44	Cortex	Male	0.244	0.070	0.418	0.006	0.081
CD44	Hippocampus	Female	0.493	−0.040	1.026	0.069	0.379
CD44	Hippocampus	Male	0.405	−0.192	1.003	0.184	0.604

### 3.3 Astrocyte-specific upregulation of CD44 in AD

**Fig. 4:**
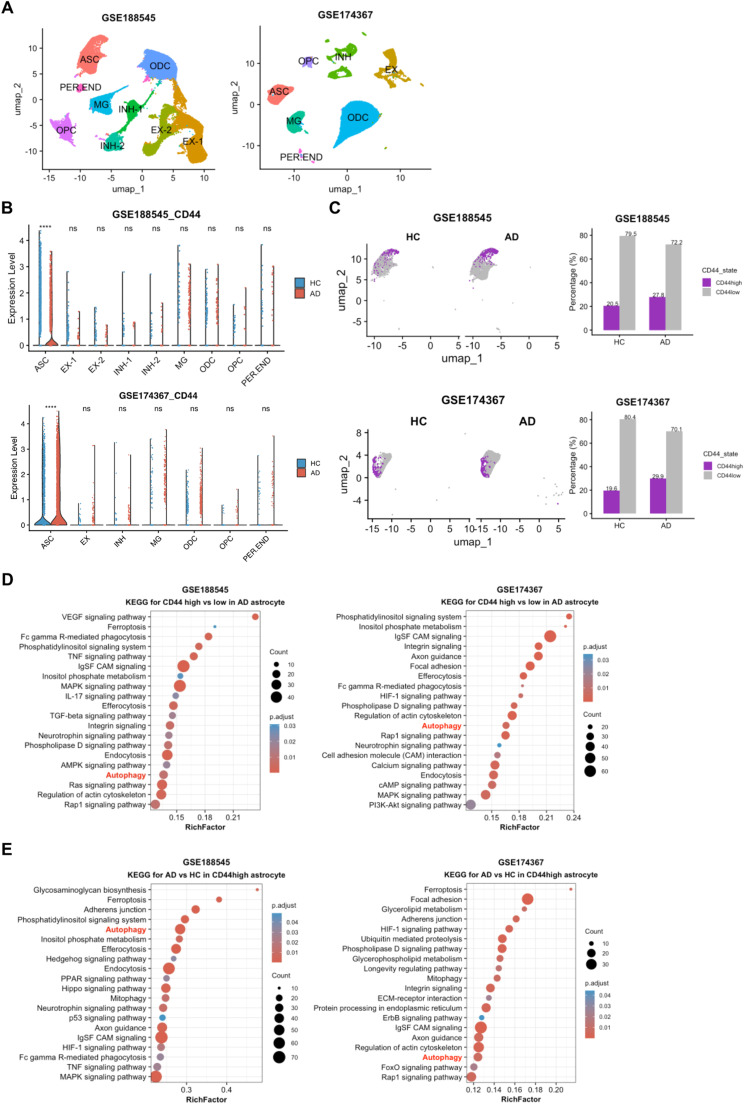
**Cellular expression of CD44 in human brain snRNA-seq datasets. A** UMAP visualization of cell clusters in snRNA-seq datasets GSE188545 and GSE174367. **B** Violin plots of CD44 expression across cell types. **C** UMAP plots of CD44-high (purple) and CD44-low (grey) astrocytes. Bar plots showed the percentages of CD44-high (purple) and CD44-low (grey) astrocytes. **D** KEGG pathway enrichment of DEGs for comparison between CD44-high and CD44-low astrocytes in AD group. **E** KEGG pathway enrichment of DEGs for comparison between AD and HC in CD44-high astrocytes. Wilcoxon test was used for comparisons. Significance levels are denoted as follows: ns, not significant; *p < 0.05; **p < 0.01; ***p < 0.001; ****p < 0.001.

To elucidate the cellular origin of CD44 upregulation in AD, we analyzed two snRNA-seq datasets (GSE188545 and GSE174367) from AD and control brains (Fig. 4A). In healthy controls, CD44 expression was predominantly localized in astrocytes, with minimal expression in other neural cell types, such as microglia, endothelial cells and neuronal cells (Fig. 4B). Astrocytes from AD patients exhibited significantly higher CD44 expression compared to those from controls, supporting a notion that astrocytic CD44 plays a role in AD pathogenesis. Furthermore, we stratified astrocytes into CD44-high and CD44-low subpopulations, with the percentages of 27.8% and 29.9% for CD44-high subpopulation in datasets GSE188545 and GSE174367, respectively (Fig. 4C). KEGG analysis of DEGs for comparison between CD44-high and CD44-low subpopulations in AD group revealed significant enrichment in autophagy, endocytosis, MAPK signaling, and phospholipase D signaling in CD44-high astrocytes (Fig. 4D). KEGG analysis of DEGs for CD44-high astrocytes between AD and HC groups revealed significant enrichment in autophagy, mitophagy, ferroptosis, and adherens junction in AD group (Fig. 4E). Therefore, we inferred that CD44 is associated with autophagy function of astrocytes.

**Fig. 5:**
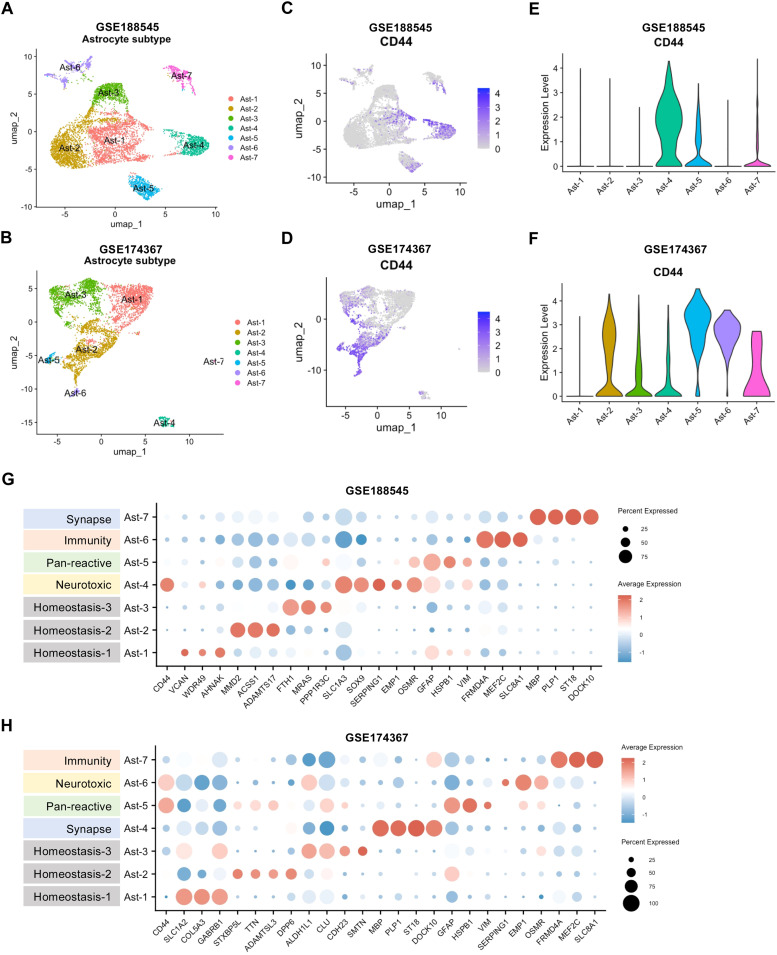
**Astrocyte subclustering and subcluster distribution of CD44. A, B** UMAP visualization of astrocyte subclustering in snRNA-seq datasets GSE188545 and GSE174367. **C, D** Scatter plots of CD44 expression across astrocyte subclusters. **E, F** Violin plots of CD44 expression across astrocyte subclusters. **E, F** Dot plots showing the expression of selected genes in astrocytes organized into homeostasis, neurotoxic, pan-reactive, immunity, and synapse clusters.

Next, we conducted a subclustering analysis of astrocytes in snRNA-seq datasets. With a resolution of 0.1, seven astrocyte subclusters were identified in both the GSE188545 and GSE174367 datasets (Supplementary Table S4). The distribution of each subcluster was visualized by UMAP (Fig. 5A, B). The scatter plot and violin plot showed that CD44 was mainly distributed in specific subpopulation of astrocytes (Fig. 5C-F). Based on the function-related genes in the previously published literature, each subcluster was annotated, such as homeostasis, immunity, neurotoxic type, pan-reactive type, and synapse. Surprisingly, we found that CD44 was mainly expressed in neurotoxic astrocytes (Fig. 5G-H), and this effect may influence the disease progression of AD.

### 3.4 CD44 as a hub gene in AD astrocytes

**Fig. 6:**
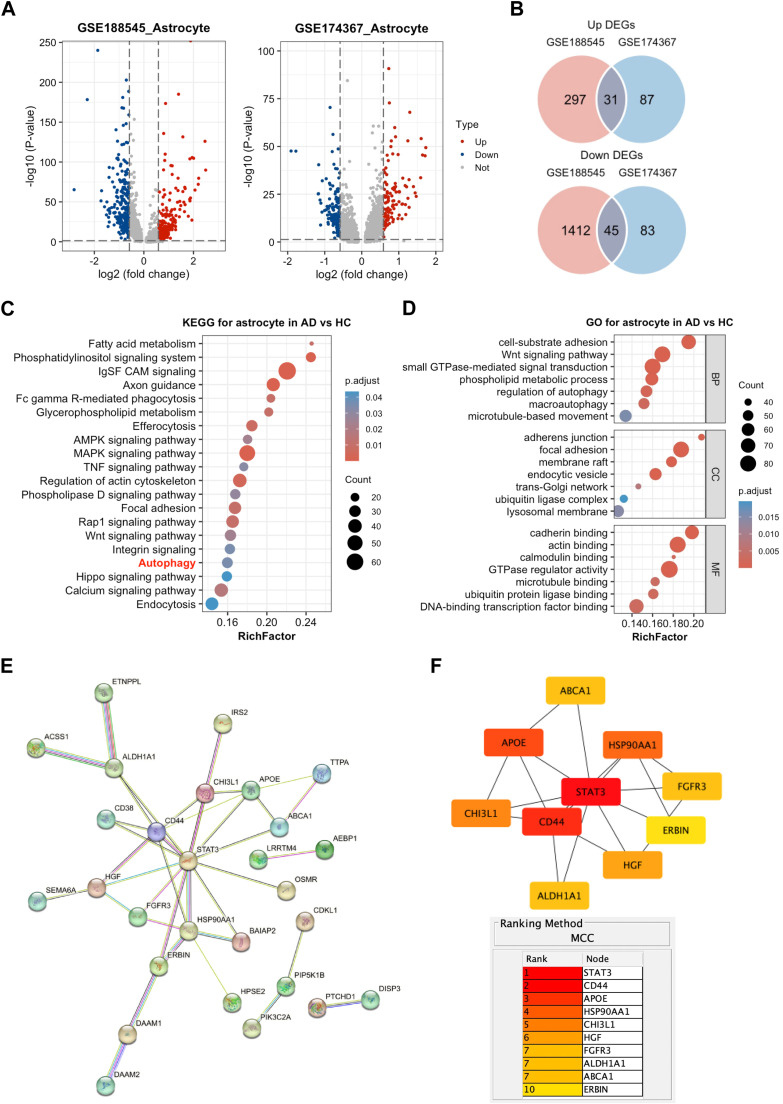
**Pathway and hub gene analysis in AD astrocytes. A** Volcano plots of astrocyte DEGs in AD vs. controls. Red, blue and gray plots represented upregulated, downregulated and not significant genes, respectively. **B** Venn diagrams of common DEGs. **C, D** Top 30 GO terms and KEGG pathways. **E** PPI network of common DEGs. **F** Top hub genes identified by CytoHubba. The descending colors from red to yellow represented declining ranking of interaction score.

To further explore the functional changes of astrocytes in AD, we investigated astrocyte-specific DEGs in AD snRNA-seq datasets (Fig. 6A). A total of 1785 DEGs (328 up, 1457 down) and 246 DEGs (118 up and 128 down) were identified in GSE188545 and GSE174367, respectively (Fig. 6B). Intersection yielded 76 common DEGs (31 up, 45 down). KEGG analysis highlighted autophagy, focal adhesion, HIF-1 signaling, and MAPK signaling (Fig. 6C), in a good agreement with the data shown in Fig. 4E. GO analysis indicated involvement in focal adhesion, synaptic transmission, and GTPase regulator activity (Fig. 6D), indicating multifaceted dysfunction in AD astrocytes. PPI network analysis revealed that DEGs in AD astrocytes form highly interconnected clusters (Fig. 6E). Using the MCC algorithm, CD44 emerged as a central hub gene within this network (Fig. 6F), suggesting that it may play an important role in astrocyte biology in the AD context. While these findings position CD44 as a potential key node in the astrocytic transcriptional response to AD pathology, they do not establish whether CD44 contributes to adaptive or non-adaptive changes. We therefore proceeded to functional experiments to directly test the role of CD44 in astrocyte autophagy.

### 3.5 Inference of SPP1 as the ligand of CD44 receptor in AD

**Fig. 7:**
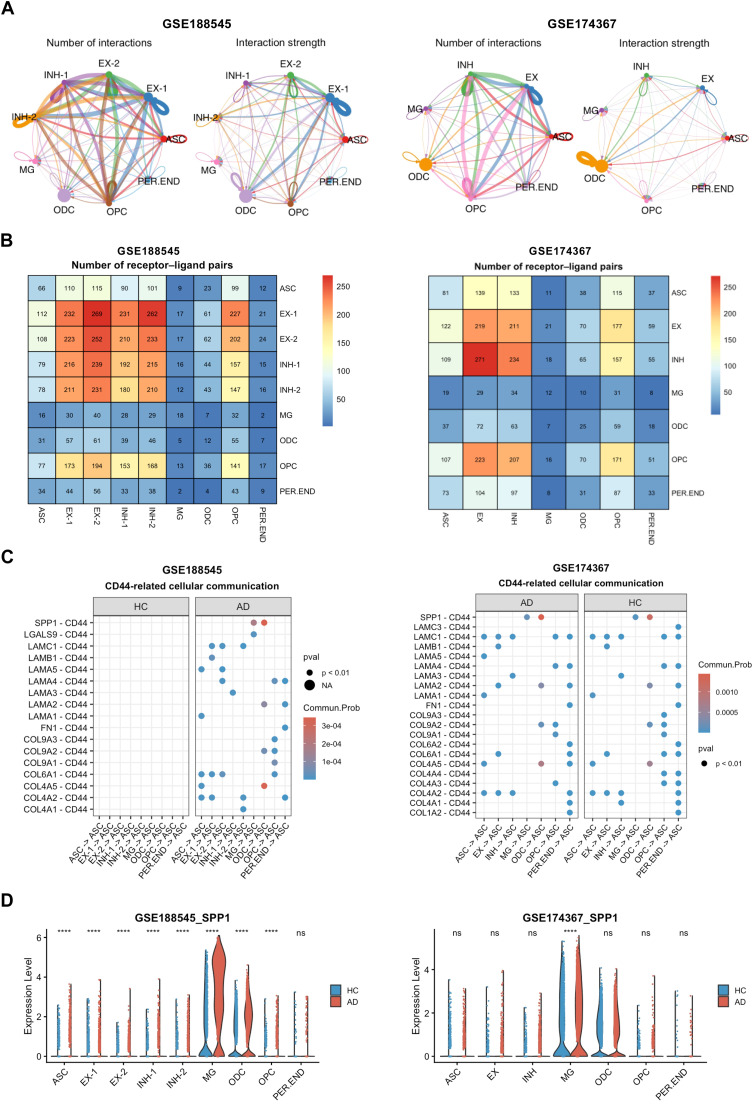
**CD44-related receptor-ligand interaction analysis. A** Communication network showing receptor-ligand interactions across cell types in snRNA-seq datasets GSE188545 and GSE174367. **B** Heatmap showing the number of receptor-ligand pairs between different cell types. **C** Bubble diagram showing the expression patterns of CD44-related receptor-ligand interaction pairs in Control and AD groups. **D** Violin plots Of SPP1 expression across cell types.

To identify potential ligands interacting with CD44 receptor in AD, we performed cell-cell communication analysis using the CellChat approach. Evaluation of receptor-ligand pair number and interaction strength revealed that intercellular communication in AD was primarily localized to astrocytes, oligodendrocytes, and oligodendrocyte progenitor cells, with astrocytes showing the most pronounced involvement (Fig. 7A, B). We next calculated the communication probabilities for all ligand-receptor interactions associated with individual signaling pathways. This analysis identified SPP1 and Collagen Type IV Alpha 5 Chain (COL4A5) as major ligands linked to CD44 receptor signaling (Fig. 7C). Notably, the communication probability for the SPP1-CD44 pair was consistently higher than that for the COL4A5-CD44 pair in both control and AD groups. The dominant SPP1-CD44 signaling axis was further confirmed by LIANA analysis (Fig. S5; Supplementary Table S5). CD44 is a well-characterized transmembrane receptor known to engage extracellular ligands, including hyaluronan (HA), osteopontin (OPN, encoded By SPP1), collagens, and matrix metalloproteinases (MMPs), to transduce signals that modulate diverse physiological and pathological processes [15]. Subsequent examination Of SPP1 expression patterns in snRNA-seq datasets revealed marked upregulation *O. spp1* in AD microglia and oligodendrocytes (Fig. 7D). In addition, analysis of HA-related gene expression showed that hyaluronan synthases (HAS1, HAS2, HAS3) and hyaluronidases (HYAL1, HYAL2, HYAL3) were expressed at very low levels, with no significant changes between groups (Fig. S2). These findings collectively suggest that CD44-SPP1 signaling contributes to enhanced cross-talk between astrocytes and other glial cell types during AD progression.

### 3.6 CD44 impairs autophagic activity in astrocytes

**Fig. 8:**
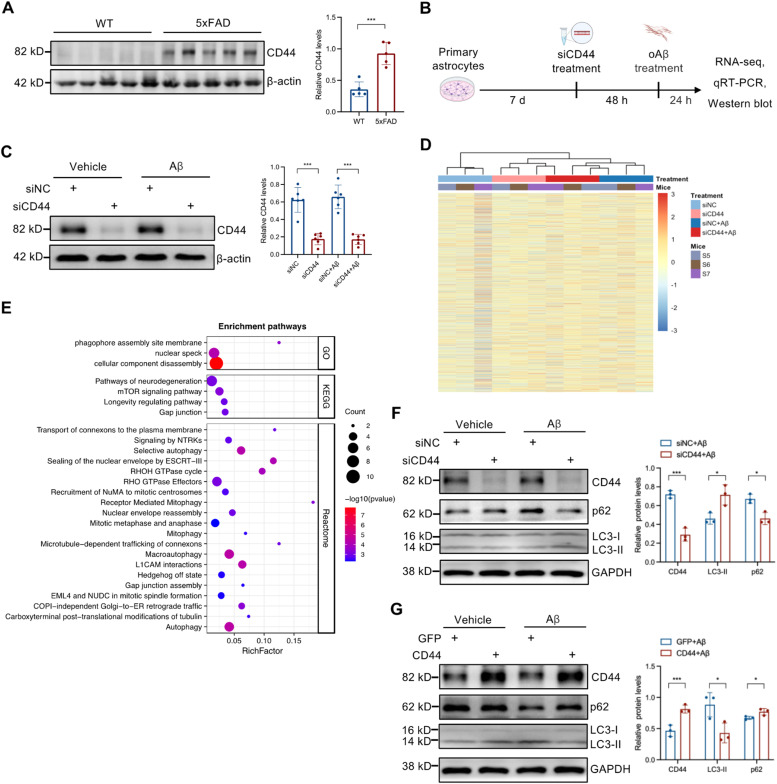
**CD44 regulates autophagy in astrocytes. A** CD44 protein levels measured in 5xFAD mouse brain by western blot (WT mice, n = 5; 5xFAD mice, n = 5). **B** Experimental workflow. Primary astrocytes were sequentially treated with siCD44 (40 nM) for 48 h and oAβ (5 μM) for 24 h, then analyzed by RNA-seq, qRT-PCR and western blot. **C** Verification of CD44 knockdown by western blot (n = 6). **D** Hierarchical clustering of detected genes across all samples (n = 3). **E** GO, KEGG and Reactome pathway enrichment for upregulated DEGs under Aβ treatment. **F, G** Western blot of LC3-II and p62 following CD44 knockdown or CD44 overexpression (n = 3). Data are presented as the mean±SD. *t*-test was used and *P*-values are shown. WT, wild type; siNC, negative control siRNA; siCD44, mouse CD44-targeted siRNA; oAβ, Amyloid-beta 42 oligomers; GO, Gene Ontology; KEGG, Kyoto Encyclopedia of Genes and Genomes.

To verify elevated CD44 expression in bioinformatics analysis, we carried out western blotting analysis in 5xFAD mice, a commonly used AD mouse model [37]. There was a marked increase in levels of CD44 in the cortex of 9-month-old 5xFAD mice compared to age-matched wild-type controls (Fig. 8A). Next, we investigated the impact of CD44 deficiency on the transcriptomic change in the presence of Aβ oligomers. We performed a bulk RNA-seq on primary astrocytes following CD44 knockdown and treatment with 5 µM Aβ oligomers (Fig. S3A, B). Western blotting showed high efficacy of siRNA-mediated CD44 knockdown (Fig. 8B, C). Hierarchical clustering analysis showed that transcriptional profiles clustered primarily by Aβ treatment (Fig. 8D). Aβ treatment induced an A1-like neurotoxic phenotype of astrocytes (Fig. S3C), as evidenced by altered expression of established pan-reactive, A1-specific, and A2-specific astrocyte markers [38,39]. Moreover, DEG analysis was conducted for CD44 knockdown under both basal and Aβ-treated conditions (siCD44 vs siNC, siCD44+Aβ vs siNC+Aβ) (Fig. S3D). CD44 knockdown under basal condition resulted in 736 DEGs (5.34% of detected genes), whereas under Aβ treatment, only 134 DEGs (1.06% of detected genes) were identified (Fig. S3E). A core set of 61 DEGs (29 up, 32 down) was common to both conditions (Fig. S3F), though these were not enriched in any KEGG pathway.

Under Aβ treatment, CD44 knockdown resulted in 54 upregulated DEGs compared to control siRNA-treated astrocytes. Pathway enrichment analysis revealed that these upregulated genes were significantly associated with several biological processes, including autophagy, mTOR signaling, gap junction organization, and cellular component disassembly (Fig. 8E). CD44 knockdown upregulated these DEGs under Aβ treatment conditions, but the exact mechanism underlying this association cannot be inferred from this statement alone. No significant pathway was enriched among the downregulated DEGs. Among autophagy-related DEGs, Tubb4b and Atg12 mRNA levels were upregulated upon CD44 knockdown (Fig. S4). Moreover, CD44 knockdown promoted autophagic activation in astrocytes, as evidenced by increased LC3-II and decreased p62 levels (Fig. 8F). Conversely, CD44 overexpression reduced LC3-II and increased p62 levels (Fig. 8G), suggesting that CD44 knockdown activates autophagy based on the levels of LC3-II and p62. Together, these results demonstrate that CD44 alteration modulates astrocytic autophagic activity under AD-related stress.

## 4 Discussion

The complex orchestration of glial responses and proteostatic failure remains a focal point in understanding AD progression. In this study, we integrated multi-cohort transcriptomic data and single-nucleus resolution to identify CD44 as a critical hub gene in the AD brain. Our findings demonstrate that CD44 is specifically upregulated in astrocytes and acts as a molecular brake on autophagic activity. By modulating the CD44-SPP1 signaling axis, astrocytes may transition toward a neurotoxic phenotype, thereby exacerbating the amyloidogenic environment.

Here, we identified CD44, a transmembrane glycoprotein receptor as a consistently and highly upregulated gene in key AD brain regions. In the mammalian cortex, CD44 is predominantly expressed in fibrous astrocytes, while protoplasmic astrocytes upregulate it under pathological conditions such as hypoxia and seizures [40]. Previous bioinformatic studies have linked CD44 to immune response [41], neuroinflammation [42], ferroptosis [43], energy metabolism [44], and extracellular matrix [45] in AD. Moreover, single-nucleus RNA sequencing (snRNA-seq) studies have consistently reported elevated CD44 expression specifically in AD astrocytes [[46], [47], [48], [49]]. Nevertheless, the mechanistic contribution of CD44 to AD pathogenesis remains poorly defined. CD44 upregulation in AD brain was further supported by re-analysis of NeuroPro database, a comprehensive resource integrating protein-level changes from 38 CE proteomic studies across 13 human brain regions [50]. CD44, along with GFAP, APP, HSPB1, and CLU, was among the most consistently upregulated proteins in NeuroPro database, while VGF, RPH3A, CORO1A, ACTN2, and HOMER1 were among the most downregulated proteins in AD brain. The concordant upregulation of CD44 at both transcript and protein levels underscores its potential as a novel biomarker and therapeutic target in AD. Strikingly, CD44 upregulation occurs during the preclinical stage of AD but does not correlate with dementia severity. Besides, the NeuroPro resource has identified 64 proteins, including APOE, AQP4, MAOB, and CD44, but not APP or MAPT, whose expression levels were altered prior to the appearance of classical neuropathology and throughout disease progression [51]. Besides, a separate large-scale proteomic study of AD brain and cerebrospinal fluid also reported early alterations in energy metabolism linked to glial activation, with CD44 significantly elevated across both asymptomatic and symptomatic AD stages [44]. Thus, CD44 may contribute to AD-associated pathology.

We further demonstrate that CD44 modulates autophagy in astrocytes during AD. There is accumulating evidence that CD44 can suppress autophagy in various cell types, such as tumor cells, vascular endothelial cells, chondrocytes, and erythroblasts [[52], [53], [54]]. In AD, astrocytes undergo dynamic alterations in autophagic activity, which plays a critical role in clearing toxic protein aggregates such as Aβ. Thus, targeting astrocytic autophagy has emerged as a potential therapeutic strategy [55]. Our data show that CD44 was upregulated specifically in astrocytes and that its knockdown rescued Aβ-induced autophagy impairment. However, the role of CD44 in neurological disorders is context-dependent, with reported neuroprotective and neurotoxic effects likely due to alternative splicing or ligand interactions [16,56]. For instance, CD44 loss exacerbated degeneration in a retinitis pigmentosa model [57], but improved outcomes in Parkinson's disease and epilepsy models [58,59]. In hippocampal neurons, CD44 overexpression was neuroprotective [60], while in neural stem cells, it promoted quiescence [61]. These divergent roles underscore the need to clarify context-specific functions of CD44 and its ligands in AD. Importantly, investigation of astrocytic CD44 is also required for further understanding of its pathological function and regulation in the context of various brain disorders.

Given the well-established sex differences in AD epidemiology, that is, female having higher prevalence and mortality than male [62], we examined whether CD44 expression differs by sex in our datasets. In a meta-analysis of bulk transcriptomic datasets, CD44 upregulation in AD was significant in cortex of females, but not in that of males. However, in our bulk transcriptomic datasets, CD44 expression in AD was higher in both males and females. Besides, a higher baseline of CD44 expression in female was observed compared to male in both AD and control groups, which may contribute to the elevated AD risk in females if this represents a primed status for astrocyte reactivity or neurotoxicity. Mechanistically, estrogen response elements have been identified in the CD44 promoter region, and influence CD44 splicing and function [63]. Interestingly, sex differences modulate several physiological and molecular processes, such as inflammation, autophagy, and metabolism, which may explain the observed sex disparities in AD [64]. Future studies are needed to definitively establish whether CD44-mediated autophagy dysfunction contributes to sex differences in AD pathology.

Our findings identified SPP1 as the top-ranked ligand in CD44-mediated intercellular communication in AD. OPN is a multifunctional phosphoprotein predominantly secreted by pro-inflammatory microglia and macrophages in neurodegenerative contexts [65]. Its selective upregulation in AD-associated myeloid cells suggests a role in neuroinflammation-glia crosstalk. The mechanistic link between SPP1-CD44 signaling and autophagy modulation warrants further investigation. CD44 is a transmembrane glycoprotein that lacks intrinsic kinase activity but signals through association with adaptor proteins and cytoskeletal linkers. Notably, Proteomic studies have identified moesin (MSN), a protein containing a four-point-one, ezrin, radixin, moesin (FERM) domain, along with the receptor CD44, as central hub proteins strongly associated with AD traits [66]. The intracellular domain of CD44 contains binding sites for FERM domain, and inhibiting the FERM-CD44 interaction helps limit AD-associated neuronal damage [67]. FERM proteins have been implicated in autophagosome formation and trafficking through their interactions with the autophagy mechanism [68]. Hence, we hypothesize that OPN binding to CD44 may induce conformational changes that alter FERM recruitment, thereby affecting the cytoskeletal reorganization required for autophagosome-lysosome fusion.

Several limitations of this study should be acknowledged. We were unable to directly compare CD44 expression between asymptomatic AD and mild cognitive impairment (MCI) cohorts. It also remains unclear whether CD44 upregulation is a cause or consequence of AD pathology. We did not perform experiments applying autophagy agonists or inhibitors, to confirm our conclusion about the influence of CD44 on astrocytic autophagy. While we established a link between CD44 and autophagy, the precise intracellular signaling intermediate (e.g., PI3K/Akt or mTOR pathways) through which CD44 modulates the autophagic machinery requires further elucidation. Additionally, although we validated CD44′s role in vitro, further studies employing astrocyte-specific conditional knockout models in AD mice will be essential to define its contributions to disease progression.

## 5 Conclusion

In conclusion, our study identifies elevated CD44 across neuroanatomical regions in AD. We demonstrate a CD44-dependent control of astrocytic autophagic activity and propose that the SPP1/CD44 signaling axis may mediate microglia-astrocyte communication. Further investigation of this pathway may yield novel strategies for AD treatment.

## Declaration of generative AI and AI-assisted technologies in the writing process

No artificial intelligence tools were used in the conceptualization, data analysis, or drafting of this manuscript. Gemini was employed solely for minor language editing after the manuscript was completed, contributing to approximately 5 % of the final content refinement.

## Ethics statement

The animal experiments were approved by the animal ethics committee of Zhongshan Hospital, Fudan University. All procedures were performed in compliance with the relevant guidelines and regulations.

## Data availability

The datasets and custom code generated and/or analyzed during the current study are available from the corresponding author upon reasonable request.

## Funding sources

This study was supported by the National Natural Science Foundation of China (82171408 to C.Z. and 82472327 to Y.T.), the Shanghai Municipal Science and Technology Major Project, and the Health Commission of Jinshan District, Shanghai (JSKJ-KTQN-2022-05 to L.D.).

## CRediT authorship contribution statement

**Haiyan Wang:** Writing – review & editing, Methodology, Software, Investigation, Formal analysis, Visualization, Data curation. **Ying Long:** Writing – original draft, Methodology, Investigation, Formal analysis, Data curation. **Yu Tang:** Writing – review & editing, Supervision, Funding acquisition. **Lijie Duan:** Resources, Funding acquisition. **Zijie Wang:** Resources. **Shuzhen Zhang:** Resources. **Yanqing Yin:** Resources. **Jiawei Zhou:** Writing – review & editing, Supervision. **Wenjuan Wu:** Writing – review & editing, Supervision, Project administration. **Chunjiu Zhong:** Writing – review & editing, Conceptualization, Supervision, Project administration, Funding acquisition.

## Declaration of competing interest

The authors declare the following financial interests/personal relationships which may be considered as potential competing interests: Yu Tang reports financial support was provided by National Natural Science Foundation of China. Chunjiu Zhong reports financial support was provided by National Natural Science Foundation of China. Chunjiu Zhong reports financial support was provided by Shanghai Municipal Science and Technology Major Project. Lijie Duan reports financial support was provided by Health Commission of Jinshan District, Shanghai. If there are other authors, they declare that they have no known competing financial interests or personal relationships that could have appeared to influence the work reported in this paper.

## Appendix Supplementary materials

Image, application 1

## References

[1] Jack C.R., Bennett D.A., Blennow K., Carrillo M.C., Dunn B., Haeberlein S.B. (2018). NIA-AA Research Framework: toward a biological definition of Alzheimer's disease. Alzheimers Dement.

[2] Jack C.R., Andrews J.S., Beach T.G., Buracchio T., Dunn B., Graf A. (2024). Revised criteria for diagnosis and staging of Alzheimer's disease: alzheimer's association workgroup. Alzheimers Dement.

[3] Alzheimer’s Association (2024). 2024 Alzheimer’s disease facts and figures. Alzheimers Dement.

[4] Liu X., Chen S., Zhang D., Gu Y., Li G., Wu B. (2025). Projected prevalence and economic burden of alzheimer's disease and related dementias in china: regional disparities and policy implications. Health Data Sci.

[5] Zhang J., Zhang Y., Wang J., Xia Y., Zhang J., Chen L. (2024). Recent advances in Alzheimer's disease: mechanisms, clinical trials and new drug development strategies. Signal Transduct Target Ther.

[6] van Dyck C.H., Swanson C.J., Aisen P., Bateman R.J., Chen C., Gee M. (2023). Lecanemab in early Alzheimer's Disease. N Engl J Med.

[7] Sims J.R., Zimmer J.A., Evans C.D., Lu M., Ardayfio P., Sparks J. (2023). Donanemab in early symptomatic alzheimer disease: the TRAILBLAZER-ALZ 2 randomized clinical trial. JAMA.

[8] Jia J.P., Ning Y.Y., Chen M.L., Wang S.H., Yang H., Li F.Y. (2024). Biomarker changes during 20 years preceding Alzheimer's Disease. New Engl J Med.

[9] Hansson O., Blennow K., Zetterberg H., Dage J. (2023). Blood biomarkers for Alzheimer's disease in clinical practice and trials. Nature Aging.

[10] Rafii M.S., Aisen P.S. (2023). Detection and treatment of Alzheimer's disease in its preclinical stage. Nature Aging.

[11] Wang W.X., Lu J.C., Pan N.Y., Zhang H.Y., Dai J.C., Li J. (2024). Identification of early Alzheimer's disease subclass and signature genes based on PANoptosis genes. Front Immunol.

[12] Lian P.P., Cai X., Wang C.L., Liu K., Yang X.M., Wu Y. (2023). Identification of metabolism-related subtypes and feature genes in Alzheimer's disease. J Transl Med.

[13] Zhang Y.D., Miao Y.Y., Tan J., Chen F.L., Lei P., Zhang Q. (2023). Identification of mitochondrial related signature associated with immune microenvironment in Alzheimer's disease. J Transl Med.

[14] López-Cerdán A., Andreu Z., Hidalgo M.R., Soler-Sáez I., de la Iglesia-Vayá M., Mikozami A. (2024). An integrated approach to identifying sex-specific genes, transcription factors, and pathways relevant to Alzheimer's disease. Neurobiol Dis.

[15] Weng X., Maxwell-Warburton S., Hasib A., Ma L., Kang L. (2022). The membrane receptor CD44: novel insights into metabolism. Trends Endocrinol Metab.

[16] Zhang M., Lin Y., Wei H., Ju Q., Gao T., Zhang Y. (2025). The membrane receptor CD44: roles in neurodegenerative diseases. Expert Opin Ther Targets.

[17] Mizushima N., Levine B. (2020). Autophagy in human diseases. N Engl J Med.

[18] Singh B., Mahajan S., Abdella S., Khan R., Garg S. (2026). Exploring autophagy inducing molecules: targeting diverse pathways in Alzheimer's Disease management. Med Res Rev.

[19] Lee J.H., Chang W., Min S.S., Song D.Y., Yoo H.I (2025). Beyond support cells: astrocytic autophagy as a central regulator of cns homeostasis and neurodegenerative diseases. Cells.

[20] Kotani T., Nakatogawa H. (2026). Core principles of autophagy initiation mechanisms. Nat Struct Mol Biol.

[21] Ritchie M.E., Phipson B., Wu D., Hu Y.F., Law C.W., Shi W. (2015). Limma powers differential expression analyses for RNA-sequencing and microarray studies. Nucleic Acids Res.

[22] Conway J.R., Lex A., Gehlenborg N. (2017). UpSetR: an R package for the visualization of intersecting sets and their properties. Bioinformatics.

[23] Yu G.C., Wang L.G., Han Y.Y. (2012). He QY. clusterprofiler: an r package for comparing biological themes among gene clusters. Omics-J Integr Biol.

[24] Yu G.C., He Q.Y. (2016). ReactomePA: an R/Bioconductor package for reactome pathway analysis and visualization. Mol Biosyst.

[25] Majeed A., Mukhtar S. (2023). Protein-protein interaction network exploration using cytoscape. Methods Mol Biol.

[26] Chin C.H., Chen S.H., Wu H.H., Ho C.W., Ko M.T., Lin C.Y. (2014). CytoHubba: identifying hub objects and sub-networks from complex interactome. BMC Syst Biol.

[27] Xu M., Zhang D.F., Luo R.C., Wu Y., Zhou H.J., Kong L.L. (2018). A systematic integrated analysis of brain expression profiles reveals YAP1 and other prioritized hub genes as important upstream regulators in Alzheimer's disease. Alzheimers & Dementia.

[28] Habib N., McCabe C., Medina S., Varshavsky M., Kitsberg D., Dvir-Szternfeld R. (2020). Disease-associated astrocytes in Alzheimer's disease and aging. Nat Neurosci.

[29] Li Y., Pereda Serras C., Blumenfeld J., Xie M., Hao Y., Deng E. (2025). Cell-type-directed network-correcting combination therapy for Alzheimer's disease. Cell.

[30] Dimitrov D., Turei D., Garrido-Rodriguez M., Burmedi P.L., Nagai J.S., Boys C. (2022). Comparison of methods and resources for cell-cell communication inference from single-cell RNA-Seq data. Nat Commun.

[31] Zhang L.S., Xu Z.Z., Jia Z.H., Cai S.C., Wu Q., Liu X.Y. (2025). Modulating mTOR-dependent astrocyte substate transitions to alleviate neurodegeneration. Nature Aging.

[32] Minhas P.S., Jones J.R., Latif-Hernandez A., Sugiura Y., Durairaj A.S., Wang Q. (2024). Restoring hippocampal glucose metabolism rescues cognition across Alzheimer's disease pathologies. Science.

[33] Chen Q., Guo X., Wang H., Sun S., Jiang H., Zhang P. (2024). Plasma-free blood as a potential alternative to whole blood for transcriptomic analysis. Phenomics.

[34] Kim D., Paggi J.M., Park C., Bennett C., Salzberg S.L. (2019). Graph-based genome alignment and genotyping with HISAT2 and HISAT-genotype. Nat Biotechnol.

[35] Li H., Handsaker B., Wysoker A., Fennell T., Ruan J., Homer N. (2009). The sequence alignment/Map format and SAMtools. Bioinformatics.

[36] Pertea M., Pertea G.M., Antonescu C.M., Chang T.C., Mendell J.T., Salzberg S.L. (2015). StringTie enables improved reconstruction of a transcriptome from RNA-seq reads. Nat Biotechnol.

[37] Forner S., Kawauchi S., Balderrama-Gutierrez G., Kramar E.A., Matheos D.P., Phan J. (2021). Systematic phenotyping and characterization of the 5xFAD mouse model of Alzheimer's disease. Sci Data.

[38] Liddelow S.A., Guttenplan K.A., Clarke L.E., Bennett F.C., Bohlen C.J., Schirmer L. (2017). Neurotoxic reactive astrocytes are induced by activated microglia. Nature.

[39] Cameron E.G., Nahmou M., Toth A.B., Heo L., Tanasa B., Dalal R. (2024). A molecular switch for neuroprotective astrocyte reactivity. Nature.

[40] Al-Dalahmah O., Sosunov A.A., Sun Y., Liu Y., Madden N., Connolly E.S. (2024). The matrix receptor CD44 is present in astrocytes throughout the human central nervous system and accumulates in hypoxia and seizures. Cells.

[41] Yang F., Zhang N., Ou G.Y., Xu S.W. (2024). Integrated bioinformatic analysis and validation identifies immune microenvironment-related potential biomarkers in Alzheimer's Disease. Jpad-J Prev Alzheim.

[42] Wu Y., Liang S.L., Zhu H., Zhu Y.P. (2021). Analysis of immune-related key genes in Alzheimer's disease. Bioengineered.

[43] Sun Y.T., Xiao Y., Tang Q., Chen W., Lin L. (2024). Genetic markers associated with ferroptosis in Alzheimer's disease. Front Aging Neurosci.

[44] Johnson E.C.B., Dammer E.B., Duong D.M., Ping L.Y., Zhou M.T., Yin L.M. (2020). Large-scale proteomic analysis of Alzheimer's disease brain and cerebrospinal fluid reveals early changes in energy metabolism associated with microglia and astrocyte activation. Nat Med.

[45] Das S., Li Z.Z., Noori A., Hyman B.T. (2020). Serrano-Pozo A. Meta-analysis of mouse transcriptomic studies supports a context-dependent astrocyte reaction in acute CNS injury versus neurodegeneration. J Neuroinflamm.

[46] Morabito S., Miyoshi E., Michael N., Shahin S., Martini A.C., Head E. (2021). Single-nucleus chromatin accessibility and transcriptomic characterization of Alzheimer's disease. Nat Genet.

[47] Leng K., Li E., Eser R., Piergies A., Sit R., Tan M.C. (2021). Molecular characterization of selectively vulnerable neurons in Alzheimer's disease. Nat Neurosci.

[48] Cain A., Taga M., McCabe C., Green G.S., Hekselman I., White C.C. (2023). Multicellular communities are perturbed in the aging human brain and Alzheimer's disease. Nat Neurosci.

[49] Mathys H., Boix C.A., Akay L.A., Xia Z.T., Davila-Velderrain J., Ng A.P. (2024). Single-cell multiregion dissection of Alzheimer's disease. Nature.

[50] Askenazi M., Kavanagh T., Pires G., Ueberheide B., Wisniewski T., Drummond E. (2023). Compilation of reported protein changes in the brain in Alzheimer's disease. Nat Commun.

[51] Yanova M., Stepanova E., Maltseva D., Tonevitsky A. (2025). CD44 variant exons induce chemoresistance by modulating cell death pathways. Front Cell Dev Biol.

[52] Zhang L., Yang P.C., Chen J.X., Chen Z.Q., Liu Z.H., Feng G.Q. (2023). CD44 connects autophagy decline and ageing in the vascular endothelium. Nat Commun.

[53] Bai R.J., Liu D., Li Y.S., Tian J., Yu D.J., Li H.Z. (2022). OPN inhibits autophagy through CD44, integrin and the MAPK pathway in osteoarthritic chondrocytes. Front Endocrinol.

[54] Yang R., Yu D.L., Ren J.Q., Yin J.Y., Li J., Liu X.H. (2025). CD44 deficiency induces combinatory NRF2 inhibition and endoplasmic reticulum stress-associated dyserythropoiesis. Faseb J.

[55] Kim S., Chun H., Kim Y., Kim Y., Park U., Chu J. (2024). Astrocytic autophagy plasticity modulates Aβ clearance and cognitive function in Alzheimer's disease. Mol Neurodegener.

[56] Pinnera E., Gruper Y., Ben Zimra M., Kristt D., Laudon M., Naor D. (2017). CD44 splice variants as potential players in alzheimer's disease pathology. J Alzheimers Dis.

[57] Ayten M., Straub T., Kaplan L., Hauck S.M., Grosche A., Koch S.F. (2024). CD44 signaling in Müller cells impacts photoreceptor function and survival in healthy and diseased retinas. J Neuroinflamm.

[58] Wang Y.J., Li L., Wu Y.T., Zhang S.P., Ju Q.Q., Yang Y.N. (2022). CD44 deficiency represses neuroinflammation and rescues dopaminergic neurons in a mouse model of Parkinson's disease. Pharmacol Res.

[59] Kruk P.K., Nader K., Skupien-Jaroszek A., Wójtowicz T., Buszka A., Olech-Kochanczyk G. (2023). Astrocytic CD44 deficiency reduces the severity of kainate-induced epilepsy. Cells.

[60] Kim S.H., Cho Y.S., Kim Y., Park J., Yoo S.M., Gwak J. (2023). Endolysosomal impairment by binding of amyloid beta or MAPT/Tau to V-ATPase and rescue via the HYAL-CD44 axis in Alzheimer disease. Autophagy.

[61] Su W.P., Foster S.C., Xing R.B., Feistel K., Olsen R.H.J., Acevedo S.F. (2017). CD44 transmembrane receptor and hyaluronan regulate adult hippocampal neural stem cell quiescence and differentiation. J Biol Chem.

[62] Mielke M.M., Aggarwal N.T., Vila-Castelar C., Agarwal P., Arenaza-Urquijo E.M., Brett B. (2022). Consideration of sex and gender in Alzheimer's disease and related disorders from a global perspective. Alzheimers Dement.

[63] Auboeuf D., Honig A., Berget S.M., O'Malley B.W. (2002). Coordinate regulation of transcription and splicing by steroid receptor coregulators. Science.

[64] Lopez-Lee C., Torres E.R.S., Carling G., Gan L. (2024). Mechanisms of sex differences in Alzheimer's disease. Neuron.

[65] Shen X.L., Qiu Y.G., Wight A.E., Kim H.J., Cantor H. (2022). Definition of a mouse microglial subset that regulates neuronal development and proinflammatory responses in the brain. P Natl Acad Sci USA.

[66] Johnson E.C.B., Dammer E.B., Duong D.M., Ping L., Zhou M., Yin L. (2020). Large-scale proteomic analysis of Alzheimer's disease brain and cerebrospinal fluid reveals early changes in energy metabolism associated with microglia and astrocyte activation. Nat Med.

[67] Du Y., Bradshaw W.J., Leisner T.M., Annor-Gyamfi J.K., Qian K., Bashore F.M. (2023). Discovery of FERM domain protein-protein interaction inhibitors for MSN and CD44 as a potential therapeutic approach for Alzheimer's disease. J Biol Chem.

[68] Cheng X., Zhang P., Zhao H., Zheng H., Zheng K., Zhang H. (2023). Proteotoxic stress disrupts epithelial integrity by inducing MTOR sequestration and autophagy overactivation. Autophagy.

