# Supplementary material for: Identification of a CD44-dependent control of astrocytic autophagic activity in Alzheimer’s disease
Source: J Prev Alzheimers Dis. 2026 May 20;13(8):100601. doi: 10.1016/j.tjpad.2026.100601 (PMC13199780; doi:10.1016/j.tjpad.2026.100601)
Supplement: Supplementary file 1 [file mmc1.docx]

**Supplementary Figure Legends**

**Fig. S1 Intersection analysis of upregulated and downregulated DEGs in each brain region.** (**A**-**D**) Upset plots visualizing the intersection of upregulated DEGs across multiple datasets from the (**A**) temporal cortex, (**B**) frontal cortex, (**C**) entorhinal cortex, and (**D**) hippocampus. (**E**-**H**) Upset plots visualizing downregulated DEGs across multiple datasets from the (**E**) temporal cortex, (**F**) frontal cortex, (**G**) entorhinal cortex, and (**H**) hippocampus.

**Fig. S2 Expression profiling of CD44-related ligand genes across neural cell types.** Violin plots displaying the expression levels of hyaluronan synthase genes HAS1 (**A**), HAS2 (**B**), HAS3 (**C**) and hyaluronidase genes HYAL1 (**D**), HYAL2 (**E**), HYAL3 (**F**) across cell types in snRNA-seq datasets GSE188545 and GSE174367. Wilcoxon test was used for comparisons of gene expression between AD and control groups for each cell type. Statistical significance was assessed using Wilcoxon test. Significance levels are denoted as follows: ns, not significant; **p* < 0.05; ***p* < 0.01; ****p* < 0.001; *****p* < 0.0001.

**Fig. S3 DEG analysis of CD44-knockdown astrocytes under Aβ treatment.** (**A**) Cell viability of primary astrocytes treated with gradient concentration of Aβ oligomers using CCK8 assay. (**B**) Protein levels of IL1β and TNFα in primary astrocytes treated with 5 μM of Aβ oligomers using WB. (**C**) Heatmap showing the expression of pan-reactive, A1-specific, and A2-specific astrocyte markers in response to CD44 knockdown under Aβ stimulus. (**D**) Volcano plots of DEGs for comparisons between siCD44 and siNC groups, siCD44+ Aβ and siNC+Aβ groups. (**E**) The percentages of up-regulated and down-regulated DEGs for comparisons between siCD44 and siNC groups, siCD44+ Aβ and siNC+Aβ groups. (**F**) Venn diagram of up-regulated and down-regulated DEGs in comparisons of siCD44 and siNC groups, siCD44+ Aβ and siNC+Aβ groups, respectively.

**Fig. S4 Validation of autophagy-related gene expression following CD44 knockdown under Aβ stimulus.** qRT-PCR analysis of select autophagy-related DEGs identified from the RNA-seq comparison between siCD44+Aβ and siNC+Aβ treated primary astrocytes. Data are presented from six independent biological replicates. Statistical significance was assessed using Wilcoxon test. Significance levels are denoted as follows: ns, not significant; **p* < 0.05; ***p* < 0.01.

**Fig. S5 LIANA analysis for CD44-related ligand-receptor interaction.** Barplots showing the top ten smallest aggregate rank using LIANA ligand-receptor analysis in snRNA-seq datasets GSE188545 and GSE174367. The preferentially highly-ranked interactions were generated from the interaction rankings of the algorithms underlying natmi, connectome, logfc, sca, and cellphonedb. The smaller the value of aggregate rank, the higher the ranking of the ligand-receptor pair.

**Fig. S1 Intersection distribution of up-regulated and down-regulated DEGs in each tissue region.**


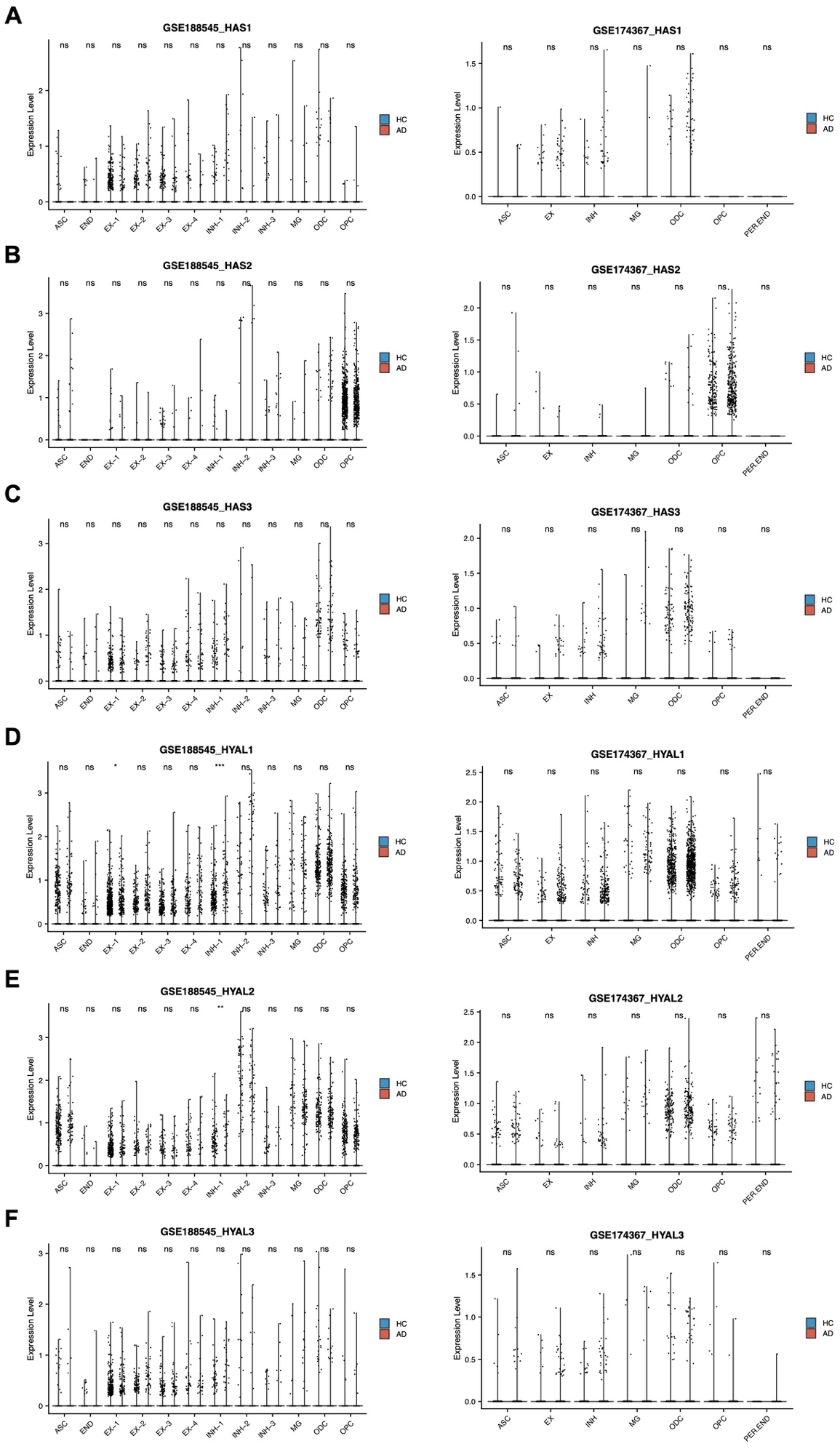


**Fig. S2 Expression patterns of hyaluronan-related genes across cell types.**

**Fig. S3 DEG analysis of CD44-knockdown astrocytes under Aβ treatment.**


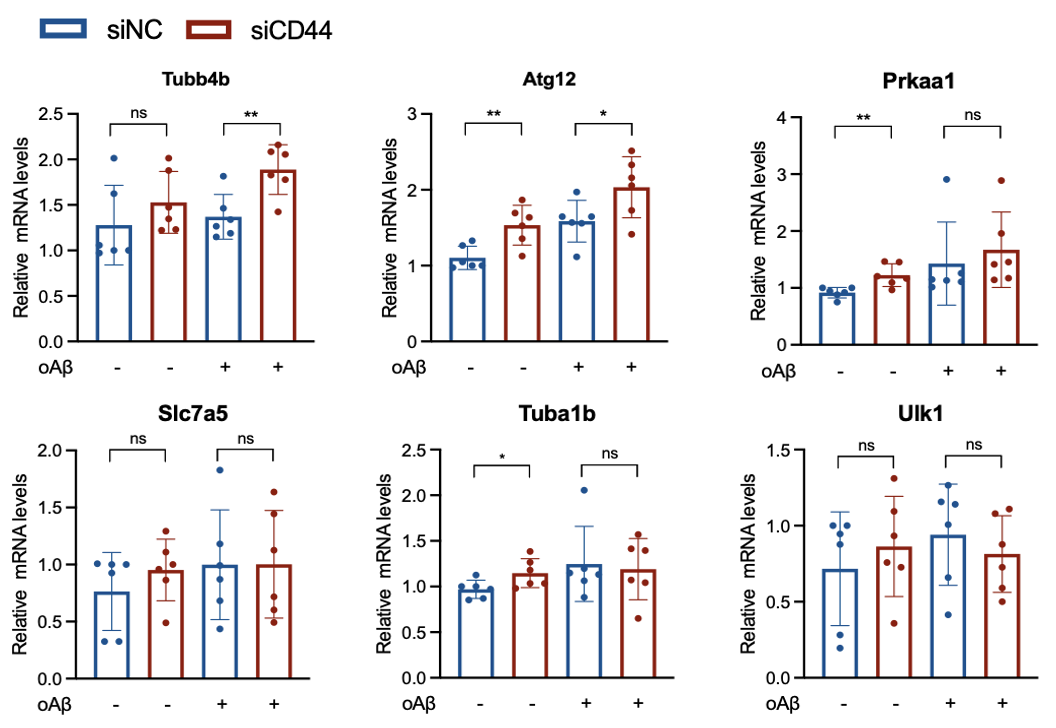


**Fig. S4 qRT-PCR analysis of autophagy-related DEGs observed between siCD44+Aβ and siNC+Aβ groups.**

**Fig. S5 LIANA analysis for CD44-related ligand-receptor interactions.**

**Supplementary Tables**

**Supplementary Table S1.** Demographic and clinicopathological characteristics of datasets analyzed in the study.

| GEO ID | Diagnosis | No. Subject | Sex  (M/F) | Age  (M (IQR)) | Braak  (M (IQR)) | MMSE  (M (IQR)) | CDR  (M (IQR)) | NFT  (M (IQR)) | Plaque score  (M (IQR)) |
| --- | --- | --- | --- | --- | --- | --- | --- | --- | --- |
| GSE29378 | Control | 16 | 11/5 | 79.0 (15.0) | 1.5 (1.0) | NA | NA | NA | 1.0 (1.0) |
|  | AD | 17 | 9/8 | 83.5 (11.8) | 5.0 (1.0) | NA | NA | NA | 3.0 (1.0) |
| GSE1297 | Control | 9 | 7/2 | 85.0 (12.0) | 2.0 (2.0) | 28.0 (3.0) | NA | 1.3 (4.4) | NA |
|  | AD | 22 | 6/16 | 85.5 (10.5) | 6.0 (1.0) | 17.0 (9.8) | NA | 21.3 (15.3) | NA |
| GSE33000 | Control | 157 | 123/34 | 62.0 (14.0) | NA | NA | NA | NA | NA |
|  | AD | 310 | 135/175 | 82.0 (12.0) | NA | NA | NA | NA | NA |
|  | HD | 157 | 83/74 | 56.0 (20.0) | NA | NA | NA | NA | NA |
| GSE132903 | Control | 98 | 50/48 | 85.0 (9.8) | NA | NA | NA | NA | NA |
|  | AD | 97 | 49/48 | 86.0 (9.0) | NA | NA | NA | NA | NA |
| GSE122063 | Control | 11 | 5/6 | 81.0 (10.5) | 0 (0) | NA | NA | NA | NA |
|  | AD | 12 | 3/9 | 81.5 (5.8) | 4.0 (2.0) | NA | NA | NA | NA |
|  | VaD | 8 | 4/4 | 80.0 (14.3) | 2.0 (2.0) | NA | NA | NA | NA |
| GSE118553 | Control | 27 | 15/12 | 75.0 (24.3) | 0 (0) | NA | NA | NA | NA |
|  | AD | 52 | 23/29 | 83.0 (14.25) | 5.0 (0) | NA | NA | NA | NA |
|  | AsymAD | 33 | 10/23 | 86.0 (10.0) | 2.0 (2.0) | NA | NA | NA | NA |
| GSE48350 | Control | 34 | 16/18 | 83.5 (17.0) | 2.0 (0.3) | 28.0 (3.0) | NA | NA | NA |
|  | AD | 28 | 11/17 | 85.5 (10.3) | 5.0 (2.0) | 12.0 (13.0) | NA | NA | NA |
|  | Young | 23 | 12/11 | 37.0 (18.0) | NA | NA | NA | NA | NA |
| GSE44772 | Control | 101 | 82/19 | 61.0 (14.0) | NA | NA | NA | NA | NA |
|  | AD | 129 | 62/67 | 82.0 (13.0) | NA | NA | NA | NA | NA |
| GSE36980 | Control | 47 | 9/9 | 80.0 (6.8) | 1.5 (1.8) | NA | NA | NA | NA |
|  | AD | 32 | 7/8 | 92.0 (8.5) | 5.0 (1.0) | NA | NA | NA | NA |
| GSE84422 | Control | 28 | 12/16 | 82.5 (16.3) | 2.0 (1.0) | NA | 0.5 (0.6) | NA | NA |
|  | AD | 47 | 14/33 | 87.0 (9.0) | 6.0 (2.0) | NA | 3.0 (3.0) | NA | NA |
|  | Possible AD | 43 | 8/35 | 89.0 (9.5) | 3.0 (1.5) | NA | 2.0 (2.0) | NA | NA |
| GSE5281 | Control | 14 | 10/4 | 79.5 (6.3) | 1.0 (0.8) | NA | NA | NA | NA |
|  | AD | 34 | 17/17 | 80.5 (10.3) | 5.0 (1.8) | NA | NA | NA | NA |
| GSE53697 | Control | 8 | NA | 79.5 (14.5) | 0 (0.3) | NA | 0 (0.5) | NA | NA |
|  | AD | 9 | NA | 93.0 (14.0) | 5.0 (1.0) | NA | 5.0 (0) | NA | NA |
| GSE125583 | Control | 70 | 37/33 | 87.0 (9.0) | 3.0 (1.0) | NA | NA | NA | NA |
|  | AD | 219 | 121/98 | 85.0 (11.0) | 5.0 (2.0) | NA | NA | NA | NA |
| GSE95587 | Control | 33 | 23/10 | 82.0 (10.0) | 3.0 (1.0) | NA | NA | NA | NA |
|  | AD | 84 | 42/42 | 87.0 (10.0) | 4.0 (1.0) | NA | NA | NA | NA |
| GSE188545 | Control | 6 | NA | NA | NA | NA | NA | NA | NA |
|  | AD | 6 | NA | NA | NA | NA | NA | NA | NA |
| GSE174367 | Control | 7 | 5/2 | 79.0 (7.5) | NA | NA | NA | 2.0 (1.0) | 1.0 (1.0) |
|  | AD | 11 | 5/6 | 89.0 (3.5) | NA | NA | NA | 6.0 (0.5) | 3.0 (1.0) |

Note: M/F, Male/Female; M (IQR), Median (interquartile range); MMSE, Mini-mental status examination; CDR, Clinical dementia rating; NFT, Neurofibrillary tangle; NA, Not available.

**Supplementary Table S2.** Non-parametric tests with ANCOVA for datasets with upregulated CD44.

| **Dataset** | **Region** | **Term** | **Sum Sq** | **F value** | **Adjusted.*p*** | **Partial η²** | **95%CI** |
| --- | --- | --- | --- | --- | --- | --- | --- |
| GSE118553 | Entorhinal cortex | (Intercept) | 33.292 | 29.965 | 1.027e-06 ^***^ |  |  |
|  |  | Disease | 20.515 | 18.465 | 6.826e-05 ^***^ | 0.355 | 0.195, 1 |
|  |  | Sex | 0.458 | 0.412 | 0.523 | 0.009 | 0.000, 1 |
|  |  | Age | 4.951 | 4.456 | 0.039 ^*^ | 0.073 | 0.002, 1 |
| GSE118553 | Frontal cortex | (Intercept) | 13.874 | 14.506 | 0.0003396 ^***^ |  |  |
|  |  | Disease | 9.738 | 10.182 | 0.002 ^**^ | 0.198 | 0.066, 1 |
|  |  | Sex | 3.274 | 3.424 | 0.069 | 0.055 | 0.000, 1 |
|  |  | Age | 0.620 | 0.648 | 0.424 | 0.011 | 0.000, 1 |
| GSE132903 | Temporal cortex | (Intercept) | 98.601 | 103.315 | < 2.2e-16 ^***^ |  |  |
|  |  | Disease | 21.876 | 22.922 | 3.374e-06 ^***^ | 0.011 | 0.048, 1 |
|  |  | Sex | 0.108 | 0.113 | 0.737 | 6.874e-04 | 0.000, 1 |
|  |  | Age | 0.010 | 0.011 | 0.917 | 5.703e-05 | 0.000, 1 |
| GSE122063 | Frontal cortex | (Intercept) | 40.952 | 38.167 | 1.566e-07 ^***^ |  |  |
|  |  | Disease | 29.799 | 27.772 | 3.534e-06 ^***^ | 0.441 | 0.263, 1 |
|  |  | Sex | 2.716 | 2.531 | 0.119 | 0.049 | 0.000, 1 |
|  |  | Age | 0.296 | 0.275 | 0.602 | 0.006 | 0.000, 1 |
| GSE122063 | Temporal cortex | (Intercept) | 18.197 | 34.150 | 4.962e-07 ^***^ |  |  |
|  |  | Disease | 11.649 | 21.862 | 2.588e-05 ^***^ | 0.403 | 0.224, 1 |
|  |  | Sex | 0.499 | 0.936 | 0.338 | 0.011 | 0.000, 1 |
|  |  | Age | 4.336 | 8.137 | 0.006 ^**^ | 0.150 | 0.027, 1 |
| GSE36980 | Hippocampus | (Intercept) | 1.270 | 1.710 | 0.212 |  |  |
|  |  | Disease | 0.017 | 0.023 | 0.882 | 0.285 | 0.016, 1 |
|  |  | Sex | 0.025 | 0.033 | 0.859 | 0.023 | 0.000, 1 |
|  |  | Age | 4.845 | 6.521 | 0.023 ^*^ | 0.318 | 0.031, 1 |
| GSE36980 | Temporal cortex | (Intercept) | 13.966 | 26.566 | 2.502e-05 ^***^ |  |  |
|  |  | Disease | 0.510 | 0.970 | 0.334 | 0.157 | 0.004, 1 |
|  |  | Sex | 0.009 | 0.017 | 0.897 | 7.008e-04 | 0.000, 1 |
|  |  | Age | 0.794 | 1.509 | 0.231 | 0.057 | 0.000, 1 |
| GSE84422 | Hippocampus | (Intercept) | 25.681 | 9.376 | 0.003 ^**^ |  |  |
|  |  | Disease | 15.651 | 5.714 | 0.019 ^*^ | 0.076 | 0.005, 1 |
|  |  | Sex | 3.961 | 1.446 | 0.234 | 0.033 | 0.000, 1 |
|  |  | Age | 0.197 | 0.072 | 0.789 | 0.001 | 0.000, 1 |
| GSE5281 | Temporal cortex | (Intercept) | 8.284 | 2.475 | 0.129 |  |  |
|  |  | Disease | 21.814 | 6.516 | 0.017 ^*^ | 0.219 | 0.026, 1 |
|  |  | Sex | 3.315 | 0.990 | 0.320 | 0.062 | 0.000, 1 |
|  |  | Age | 0.437 | 0.131 | 0.721 | 0.005 | 0.000, 1 |
| GSE5281 | Frontal cortex | (Intercept) | 14.471 | 6.080 | 0.019 ^*^ |  |  |
|  |  | Disease | 16.872 | 7.089 | 0.012 ^*^ | 0.177 | 0.019, 1 |
|  |  | Sex | 7.741 | 3.253 | 0.081 | 0.134 | 0.004, 1 |
|  |  | Age | 0.229 | 0.096 | 0.759 | 0.003 | 0.000, 1 |
| GSE5281 | Entorhinal cortex | (Intercept) | 2.451 | 1.224 | 0.282 |  |  |
|  |  | Disease | 34.257 | 17.105 | 0.001 ^***^ | 0.563 | 0.291, 1 |
|  |  | Sex | 0.023 | 0.012 | 0.915 | 0.005 | 0.000, 1 |
|  |  | Age | 3.073 | 1.534 | 0.231 | 0.075 | 0.000, 1 |
| GSE48350 | Hippocampus | (Intercept) | 18.2 | 0.162 | 0.688 |  |  |
|  |  | Disease | 53.8 | 0.479 | 0.492 | 0.090 | 0.008, 1 |
|  |  | Sex | 1081.4 | 9.630 | 0.003 ^**^ | 0.157 | 0.040, 1 |
|  |  | Age | 1224.3 | 10.903 | 0.002 ^**^ | 0.158 | 0.041, 1 |
| GSE29378 | Hippocampus | (Intercept) | 65.115 | 113.234 | 2.381e-15 ^***^ |  |  |
|  |  | Disease | 26.361 | 45.841 | 6.608e-09 ^***^ | 0.382 | 0.225, 1 |
|  |  | Sex | 1.219 | 2.120 | 0.151 | 0.059 | 0.000, 1 |
|  |  | Age | 3.983 | 6.926 | 0.010 ^*^ | 0.105 | 0.014, 1 |

Note: ^*^, adjusted *p*-value < 0.05; ^**^, adjusted *p*-value < 0.01; ^***^, adjusted *p*-value < 0.001.

**Supplementary Table S3.** Logistic regression analysis of CD44 combined with clinical characteristic variables using dataset GSE84422.

| **CDR group** | **Term** | **Estimate** | **Std.Error** | **Statistic** | **Adjusted.*p*** | **95% CI** |
| --- | --- | --- | --- | --- | --- | --- |
| CDR=0 | (Intercept) | -4.879 | 0.977 | -4.995 | 1.561e-06 | -6.808, -2.949 |
|  | CD44_state High | 0.321 | 0.170 | 1.893 | 0.060 | -0.014, 0.657 |
|  | Age | 0.087 | 0.011 | 8.097 | 1.507e-13 | 0.066, 0.108 |
|  | Sex Male | -0.034 | 0.253 | -0.135 | 0.893 | -0.534, 0.466 |
|  | Tissue_region Frontal cortex | -0.064 | 0.287 | -0.223 | 0.824 | -0.630, 0.502 |
|  | Tissue_region Temporal cortex | -0.270 | 0.286 | -0.944 | 0.347 | -0.836, 0.295 |
|  | Tissue_region Hippocampus | -0.448 | 0.345 | -1.300 | 0.196 | -1.129, 0.233 |
|  | Tissue_region Occipital cortex | 0.205 | 0.412 | 0.496 | 0.620 | -0.610, 1.019 |
|  | Tissue_region Parietal cortex | 0.031 | 0.424 | 0.073 | 0.942 | -0.807, 0.870 |
|  | Tissue_region Striatum | -0.093 | 0.358 | -0.259 | 0.796 | -0.799, 0.614 |
| CDR>0 | (Intercept) | 0.444 | 0.731 | 0.608 | 0.543 | -0.990, 1.879 |
|  | CD44_state High | 0.234 | 0.128 | 1.826 | 0.068 | -0.017, 0.485 |
|  | Age | 0.037 | 0.008 | 4.613 | 4.641e-06 | 0.021, 0.052 |
|  | SexMale | 0.130 | 0.148 | 0.877 | 0.381 | -0.161, 0.420 |
|  | Tissue_region Frontal cortex | 0.153 | 0.206 | 0.742 | 0.458 | -0.252, 0.558 |
|  | Tissue_region Temporal cortex | 0.126 | 0.212 | 0.595 | 0.552 | -0.290, 0.542 |
|  | Tissue_region Hippocampus | -0.094 | 0.244 | -0.386 | 0.699 | -0.573, 0.384 |
|  | Tissue_region Occipital cortex | 0.161 | 0.313 | 0.513 | 0.608 | -0.454, 0.775 |
|  | Tissue_region Parietal cortex | -0.311 | 0.320 | -0.971 | 0.332 | -0.939, 0.318 |
|  | Tissue_region Striatum | 0.338 | 0.251 | 1.348 | 0.178 | -0.154, 0.830 |

**Supplementary Table S4.** Top ten markers for subpopulations for astrocyte subclustering in snRNA-seq datasets.

| **GSE188545** | **Ast-1** | **Ast-2** | **Ast-3** | **Ast-4** | **Ast-5** | **Ast-6** | **Ast-7** |
| --- | --- | --- | --- | --- | --- | --- | --- |
|  | VCAN | ARL17B | HES5 | YBX3 | JUNB | MYT1L | ST18 |
|  | WDR49 | MMD2 | FTH1 | PLSCR1 | ANGPTL4 | MTUS2 | FRMD4B |
|  | AHNAK | ACSS1 | XIST | ZFP36 | HILPDA | XKR4 | KCNH8 |
|  | COL21A1 | CA12 | SRPX2 | SOCS3 | CHI3L1 | ASIC2 | PALM2 |
|  | MYBPC1 | ADAMTS17 | MRAS | TCAF2 | FOS | GALNTL6 | FA2H |
|  | CFAP54 | WIF1 | SLC6A1-AS1 | CRISPLD1 | KNOP1 | ZNF804A | MOG |
|  | AEBP1 | OLMALINC | PPP1R3C | WARS | CEBPD | HTR1E | SHROOM4 |
|  | SYTL4 | RHBDL3 | LSAMP-AS1 | RGS16 | HAP1 | SYNPR | OPALIN |
|  | GMPR | FAXDC2 | HES1 | RNF122 | DBI | MARCH11 | CD22 |
|  | RHPN1 | JAKMIP1 | ID1 | CCL2 | RASD1 | ZNF385D | FOLH1 |
| **GSE174367** | **Ast-1** | **Ast-2** | **Ast-3** | **Ast-4** | **Ast-5** | **Ast-6** | **Ast-7** |
|  | SLC1A2 | DCLK1 | HGF | ZNF536 | MYOF | CLCF1 | SLCO2B1 |
|  | CABLES1 | SLC38A1 | CHI3L1 | FA2H | PGAM2 | FAM129A | LY86 |
|  | WIF1 | STXBP5L | AC010655.4 | CD22 | GBP2 | C2CD4A | PIK3R5 |
|  | RERG | TTN | CDH23 | PALM2 | TPM4 | TEAD4 | CD84 |
|  | MFGE8 | GPC6 | SMTN | NINJ2 | ADM | FOSL1 | SIRPB2 |
|  | PIP5K1B | ADAMTSL3 | HS3ST3B1 | KLHL4 | IGFBP5 | FBLN5 | PIK3CG |
|  | CC2D2B | KCNJ3 | NQO1 | ERBB3 | GADD45A | IL1R1 | LILRB1 |
|  | SLC13A5 | PLCB4 | ADAM33 | TMEM235 | IER5L | NRP2 | TAL1 |
|  | SLC7A10 | ADAMTS9 | RIMS1 | KIRREL3 | FAS | LMO2 | TREML1 |
|  | MOXD1 | KCNJ3 | BAALC-AS1 | ST18 | LMOD1 | RGS6 | KYNU |
